# Conductive Microneedles Loaded With Polyphenol‐Engineered Exosomes Reshape Diabetic Neurovascular Niches for Chronic Wound Healing

**DOI:** 10.1002/advs.202507974

**Published:** 2025-08-26

**Authors:** Di Liu, Jingxian Gao, Xueling Wu, Xinxin Hao, Wenxiu Hu, Lu Han

**Affiliations:** ^1^ Key Laboratory of Marine Drugs, Ministry of Education, School of Medicine and Pharmacy Ocean University of China Qingdao 266003 China; ^2^ Laboratory for Marine Drugs and Bioproducts Qingdao Marine Science and Technology Center Qingdao 266237 China

**Keywords:** conductive microneedles, diabetic wound healing, exosomes, plant polyphenol

## Abstract

Diabetic wound healing remains a major clinical challenge due to the accumulation of advanced glycation end products (AGEs), reactive oxygen species (ROS), and proinflammatory cytokines under hyperglycemic conditions, which collectively impair neurovascular regeneration. Here, a biological‐electrical therapeutic platform is reported by synergizing polyphenol‐engineered *Saccharina japonica* exosomes (CA@Exos)‐derived biological signals with electroconductive microneedles (pCNTs‐ASA MNs)‐delivered electrical cues, achieving a dual‐pathway to reshape neurovascular niches during the diabetic wound healing process. CA@Exos serve as bioactive cargo to suppress AGE formation, scavenge ROS, and reverse the inflammatory microenvironment, while their intrinsic bioactivities in modulating angiogenesis and neurotrophic signaling enhanced Schwann cell‐vascular endothelial cell crosstalk. Concurrently, the conductive pCNTs‐ASA MNs functioned as spatiotemporal bioelectric scaffolds, enhancing exosome uptake and amplifying endogenous wound currents by transmitting exogenous electrical stimulation. This dual‐modality strategy synergistically promotes angiogenesis, neural regeneration, and re‐epithelialization, achieving full‐thickness wound closure in diabetic rats. This work pioneers the therapeutic potential of plant‐derived exosomes with conductive MNs‐mediated biophysical stimulation, offering a promising therapeutic strategy to disrupt the pathological feedback loop of hyperglycemic microenvironment for diabetic wound healing. The combined strategy, supported by a favorable biosafety profile and high adaptability, demonstrates a bright prospect for clinical translation, offering new hope for patients with chronic diabetic wounds.

## Graphical abstract

This study establishes a bio‐electroceutical interface by synergizing engineered exosome‐derived biological signals with electroconductive microneedle‐delivered electrical cues, achieving dual‐pathway reprogramming of the diabetic neurovascular niches and accelerating wound healing.

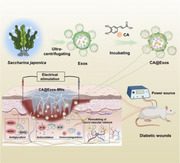

## 1 Introduction

Diabetes, a chronic condition affecting ≈537 million individuals globally, is anticipated to impact ≈643 million people by 2030.^[^ ^1^ ^]^ Among its complications, chronic diabetic wounds remain a major clinical challenge, profoundly affecting the daily lives of diabetic individuals and, in severe cases, elevating the risk of amputation.^[^ ^2^ ^]^ Persistent hyperglycemic milieu in diabetic wounds frequently leads to the excessive production of advanced glycation end products (AGEs),^[^ ^3^ ^]^ which interact with AGE receptor (RAGE), widely expressed on various cell types, including inflammatory cells, endothelial cells, and neurons.^[^ ^4^ ^]^ The AGE‐RAGE interaction continuously stimulates the production of reactive oxygen species (ROS)^[^ ^5^ ^]^ and the release of proinflammatory cytokines,^[^ ^6^ ^]^ resulting in a vicious cycle of oxidative stress and chronic inflammatory response. This cascade leads to neuronal and vascular impairments, ultimately culminating in delayed wound healing.^[^ ^7^ ^]^ Consequently, inhibiting AGE formation and mitigating the AGE‐induced diabetic angiopathy and neuropathy have been recognized as an effective strategy to promote diabetic wound repair. Although compounds such as aminoguanidine^[^ ^8^ ^]^ and pyridoxamine^[^ ^9^ ^]^ are used to inhibit AGE formation in diabetes, their clinical application is hampered by poor bioavailability and potential side effects. Furthermore, while most wound dressing studies focus on promoting angiogenesis and/or neurogenesis,^[^ ^10^ ^]^ the crucial role of the neurovascular network in diabetic wound healing is frequently underappreciated. Hence, effective inhibition of AGE formation and promotion of neurovascular network reconstruction represent promising strategies for significantly enhancing diabetic wound healing.

Caffeic acid (CA), a phenolic acid derived from potatoes, artichokes, gooseberries, and coffee,^[^ ^11^ ^]^ possesses the ability to inhibit AGE formation by obstructing protein glycation sites and sequestering intermediate glycation by‐products.^[^ ^7^, ^12^ ^]^ In addition, the catechol groups within CA exhibit robust antioxidant^[^ ^13^ ^]^ and anti‐inflammatory activities.^[^ ^7e^ ^]^ However, the poor solubility of CA significantly limits its therapeutic efficacy.^[^ ^14^ ^]^ To overcome this limitation, an effective nanocarrier is essential to improve bioavailability and therapeutic potential of CA. Recent studies have demonstrated that rationally engineered nanocarriers, such as liposomes, polymer‐based delivery systems, inorganic nanoparticles, and nanoemulsions, can significantly improve the pharmacokinetics and therapeutic efficacy of oxidative modulators (e.g., polyphenols), facilitating their precise localization, sustained release, and improved responsiveness to disease‐specific microenvironments.^[^ ^15^ ^]^ Nevertheless, these conventional nanocarriers often suffer from inherent drawbacks, including complex synthesis procedures, poor stability under physiological conditions, potential toxicity of degradation by‐products, and limited efficiency in cellular uptake.^[^ ^16^ ^]^ These limitations hinder their clinical translation and therapeutic efficacy, particularly in chronic wound scenarios that require prolonged biocompatibility and deep tissue penetration. Exosomes (Exos) have garnered significant attention as promising drug delivery vehicles,^[^ ^17^ ^]^ owing to their efficient internalization by recipient cells and their capacity to efficiently transport ribonucleic acid (RNA), thereby promoting the expression of functional proteins.^[^ ^18^ ^]^ Compared to mammalian‐derived exosomes, plant‐derived exosomes offer distinct advantages, including abundant availability, scalable production, low immunogenicity, and prolonged circulation periods, rendering them appealing alternatives for regenerative medicine and further clinical translation.^[^ ^19^ ^]^

To date, plant‐derived Exos have been demonstrated to be highly engineerable, enabling their use as versatile therapeutic vectors for various diseases.^[^ ^19^, ^20^ ^]^ For example, grapefruit‐derived Exos modified with nanoparticles encapsulating doxorubicin have shown enhanced drug delivery and significantly improved antiglioma efficacy.^[^ ^21^ ^]^ Similarly, *Polygonum multiflorum*‐derived Exos demonstrated significant anti‐photoaging effects by mitigating ultraviolet‐induced oxidative stress, inhibiting matrix metalloproteinase production, reducing extracellular matrix degradation, and promoting collagen synthesis.^[^ ^22^ ^]^ In another example, engineered ginseng‐derived small extracellular vesicles (G‐sEVs^DM^) enabled sustained didymin delivery, promoting macrophage reprogramming and inducing the differentiation of bone marrow‐derived mesenchymal stem cells into neural cells. This process established a regenerative neurogenesis‐angiogenesis cycle, ultimately facilitating a whole‐course‐repair in diabetic wounds.^[^ ^23^ ^]^ Additionally, turmeric‐derived Exos alleviated oxidative stress and restored the fibroblast‐macrophage communication network, thereby accelerating diabetic wound healing.^[^ ^24^ ^]^ *Saccharina japonica*, a brown seaweed widely used in traditional medicine and dietary supplements,^[^ ^25^ ^]^ is abundant in essential nutrients such as polysaccharides, phenolic compounds, vitamins, and minerals,^[^ ^26^ ^]^ and exhibits multiple biological functions, including antioxidant, anti‐inflammatory, angiogenic, and neuroprotective activities.^[^ ^27^ ^]^ Furthermore, the abundance of *Saccharina japonica* ensures a scalable and sustainable source of Exos, further enhancing their potential for therapeutic translation and clinical applications. However, due to their small size and high diffusivity, Exos often suffer from rapid clearance and limited penetration into deeper wound tissues. Therefore, the development of effective delivery systems is critical to prolong their retention, enhance tissue permeability, and fully realize their therapeutic efficacy in wound healing applications.

Recently, smart therapeutic platforms have emerged to overcome the delivery challenges and complex microenvironmental associated with diabetic wounds.^[^ ^28^ ^]^ For instance, a photothermal‐enhanced supramolecular hydrogel exhibited promising efficacy in treating bacteria‐infected diabetic wounds by modulating the local immune response and improving the hyperglycemic microenvironment.^[^ ^29^ ^]^ Similarly, a multifunctional self‐assembled nanocellulose‐based scaffold facilitated diabetic wound healing by offering sustained therapeutic delivery while modulating the inflammatory microenvironment and promoting tissue regeneration.^[^ ^30^ ^]^ Among these advanced platforms, microneedles have attracted particular attention as minimally invasive devices capable of painlessly penetrating the skin, enabling the delivery of bioactive agents into the deeper layers of diabetic wounds, thereby enhancing both bioavailability and therapeutic efficacy.^[^ ^31^ ^]^ For instance, piezoelectric microneedle patches have been shown to induce deep tissue cavitation and significantly enhanced drug penetration, providing a safe and minimally invasive approach for skin disease treatment.^[^ ^32^ ^]^ In addition to biochemical interventions, biophysical cues such as endogenous electric fields (EFs) play a pivotal role in synergistically promoting tissue regeneration and functional recovery at the wound site.^[^ ^33^ ^]^ Exogenous electrical stimulation (ES) has been demonstrated to magnify endogenous EF, modulating the wound microenvironment and accelerating diabetic wound recovery.^[^ ^34^ ^]^ Given the skin's sensitivity to electrical stimuli, the development of conductive microneedles capable of integrating ES emerges as a promising strategy for wound healing. Conductive microneedles not only facilitate the delivery of therapeutic agents to deeper layers of wounds but also transmit electrical signals to the wound site, consequently enhancing tissue regeneration and functional recovery.

**Figure 1:**
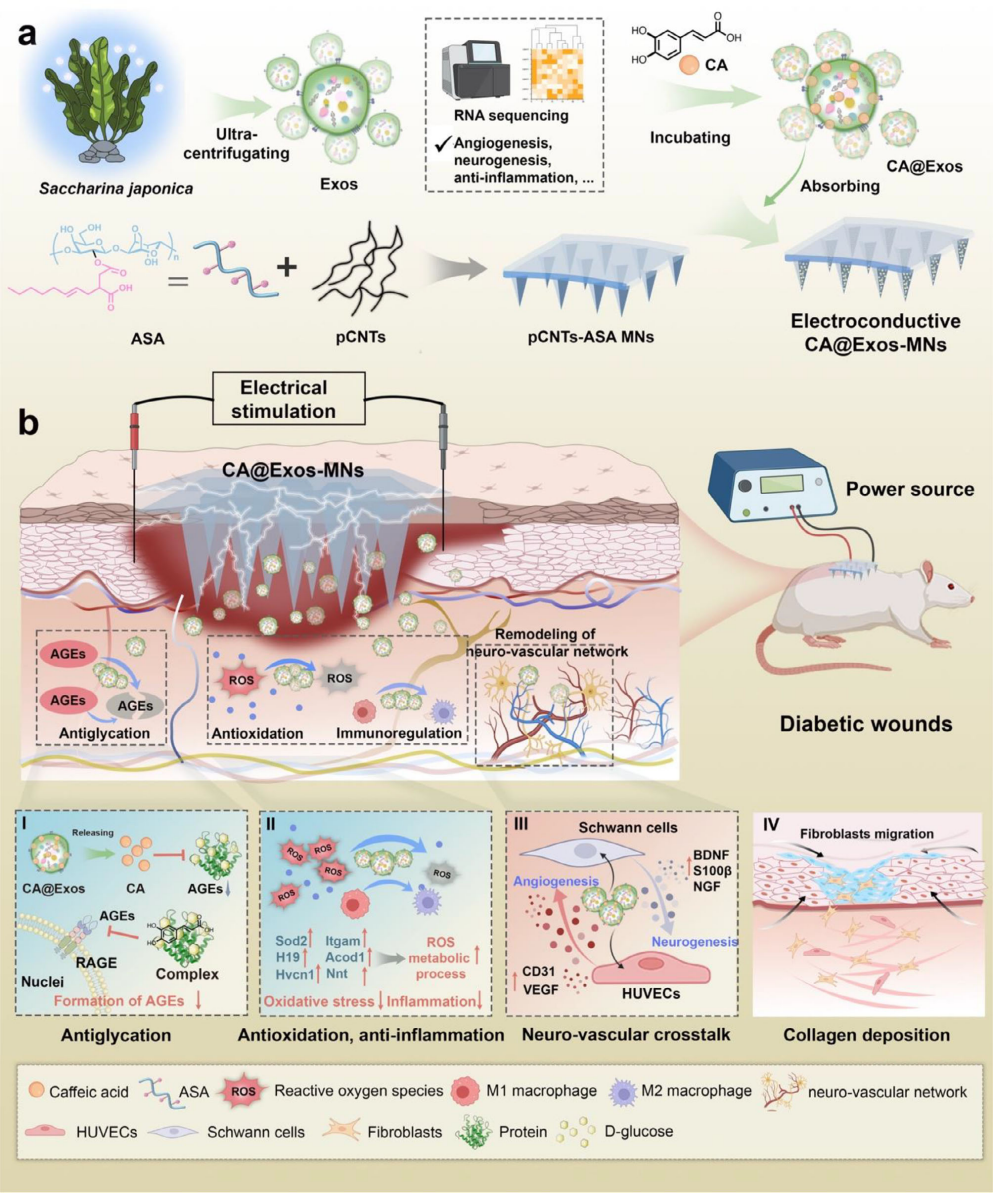
Schematic illustration of *Saccharina japonica*‐derived exosomes‐functionalized conductive microneedles to accelerate diabetic wound healing by regulating hyperglycemic microenvironment and neurovascular remodeling. a) Schematic illustration of the fabrication process, including isolation *Saccharina japonica‐*exosomes (Exos), encapsulation of CA in the Exos to obtain CA@Exos, fabrication of the conductive MNs composed of ASA and pCNTs, and physical absorption of CA@Exos onto pCNTs‐ASA MNs to obtain CA@Exos‐MNs patch. b) The combination of CA@Exos‐MNs patch and applied electrical stimulation inhibits the formation of AGEs, ROS, and inflammation, while promoting cellular migration and neurovascular communication, collagen deposition, thereby accelerating diabetic wound healing.

In this study, we developed a conductive microneedle patch loaded with polyphenol‐engineered exosomes to reshape diabetic neurovascular niches for chronic wound treatment (**Figure** **1**a). Exosomes extracted from *Saccharina japonica* were loaded with caffeic acid (CA@Exos) and physically absorbed onto the tip layer of the conductive microneedles (MNs). The conductive MNs patch, fabricated using polydopamine‐decorated carbon nanotubes (pCNTs) and octenyl succinic anhydride‐grafted agarose (ASA), exhibited sufficient mechanical strength for effective skin penetration. This design facilitated deep delivery and cellular uptake of CA@Exos via electric stimulation (ES), thereby amplifying their biological activities. Upon application to diabetic wounds, CA released from the exosomes inhibited the formation of AGEs, ROS, and proinflammatory cytokines, effectively modulating the hyperglycemic microenvironment and restoring immune homeostasis. Additionally, the conductive MNs patch facilitated the transmission of exogenous electrical stimuli, which synergistically enhanced the therapeutic effects of CA@Exos by promoting cellular migration and neurovascular communication, ultimately initiating a regenerative neurogenesis‐angiogenesis process to accelerate diabetic wound healing (Figure 1b).

## 2 Results and Discussion

### 2.1 Isolation of *Saccharina japonica*‐Derived Exosomes (Exos) and Their Bioactivity in Modulating Angiogenesis and Neurotrophic Signaling

**Figure 2:**
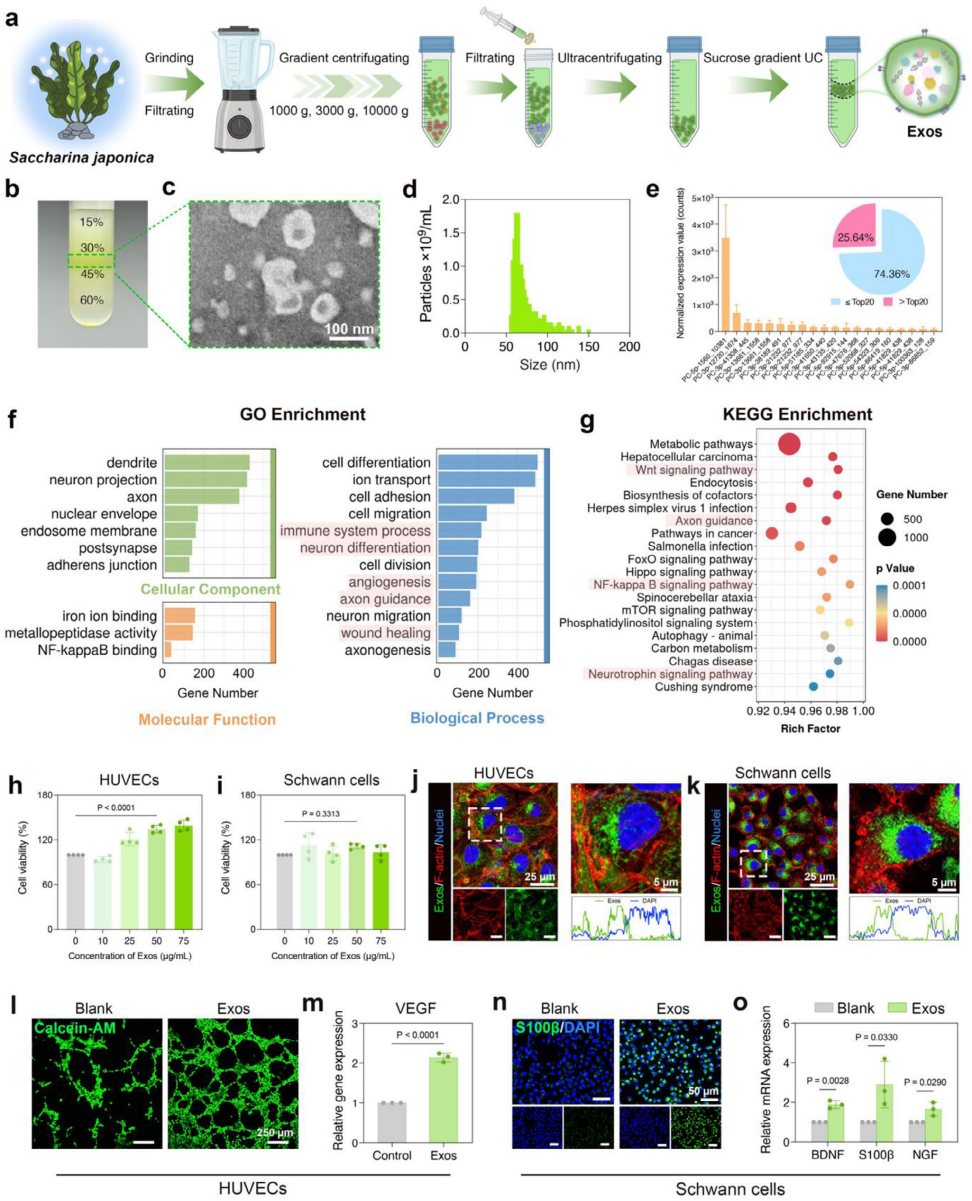
Isolation and characterization of *Saccharina japonica*‐derived exosomes. a) Schematic illustration of the isolation and purification of Exos from *Saccharina japonica*. b) Photograph of Exos bands after purification (indicated by green rectangle). c) TEM image of Exos. d) Particle size and concentration of Exos tested by NTA. e) Normalized expression values of top20 miRNAs in Exos derived from three distinct batches and the proportions (relative to all miRNAs) of top20 miRNAs in Exos. f) GO term enrichment bar graphs for biological process, cellular component, and molecular function, showing enrichment in the specific target genes of miRNAs. g) Scatterplot of KEGG pathway enrichment showing signaling pathways enriched in the specific target genes of miRNAs. h,i) Cell viability assay of HUVECs and Schwann cells co‐cultured with Exos at different concentrations (0, 10, 25, 50, and 75 µg mL^−1^) for 24 h (*n* = 4). j,k) Fluorescence visualization of Exos localization in HUVECs and Schwann cells after 6 h incubation in complete DMEM medium containing DiO‐labeled‐Exos (50 µg mL^−1^). The cell nuclei were stained using DAPI (blue), the F‐actin was stained using phalloidin (red), and Exos were labeled with DiO (green). l) Fluorescence images of Matrigel tube formation assay. HUVECs were cultured in complete DMEM medium (Blank) and complete DMEM medium containing Exos (50 µg mL^−1^) for 6 h, respectively. m) RT‐qPCR analysis of the expression levels of VEGF in HUVECs co‐cultured with Exos (50 µg mL^−1^) for 6 days (*n* = 3). n) Immunofluorescence staining of S100β (green) in Schwann cells co‐cultured with Exos (50 µg mL^−1^) for 6 days. The cell nuclei were stained using DAPI (blue). o) RT‐qPCR analysis of the expression levels of BDNF, S100β, and NGF in Schwann cells co‐cultured with Exos (50 µg mL^−1^) for 6 days (*n* = 3). Data are presented as mean values ± SD. Comparisons were performed by one‐way ANOVA followed by Tukey's multiple comparisons test in (h, i) and unpaired two‐tailed Student's *t* test in (m, o). Differences were considered statistically significant at *p* < 0.05.

*Saccharina japonica*‐derived exosomes (Exos) were first isolated and purified by a stepwise process involving differential centrifugation, ultracentrifugation, and density gradient ultracentrifugation (**Figure** **2**a). As shown in Figure 2b, the purified Exos were localized at the 30%/45% interface of the sucrose gradient. Transmission electron microscopy (TEM) image showed that the Exos displayed the typical “cup‐shaped” morphology (Figure 2c), while nanoparticle tracking analysis (NTA) quantified the concentration at 3.01 × 10^13^ particles mL^−1^ (Figure 2d). Subsequently, Exos from three distinct batches were collected and processed for microRNA (miRNA) analysis and target gene prediction. Pearson correlation analysis demonstrated a high degree of similarity among Exos derived from three distinct batches (Figure S1, Supporting Information). A length distribution of valid reads in Exos primarily concentrated at 18–25 nucleotides (Figure S2, Supporting Information), aligning with the typical length distribution observed in plant miRNAs. Furthermore, the consistency of miRNA expression profiles across the three distinct batches was confirmed, further reinforcing the reproducibility of the incorporated miRNAs within Exos (Figure 2e; Figure S3, Supporting Information). Gene Ontology (GO) term enrichment of predicted target genes revealed significant association with biological processes such as “skin development”, “wound healing”, “neuron differentiation”, “axon guidance”, “immune system process”, “angiogenesis”, and “cell migration” (Figure 2f). Additionally, a top 20 Kyoto Encyclopedia of Genes and Genomes (KEGG) enrichment pathway analysis revealed that the predicted target genes of Exos‐miRNAs were significantly enriched in angiogenesis‐related signaling pathway (Wnt signaling pathway), neural‐related signaling pathways (axon guidance and neurotrophin signaling pathway), and inflammatory‐related signaling pathways (NF‐kB signaling pathway) (Figure 2g). Both GO and KEGG analysis results demonstrate the considerable potential of Exos in modulating angiogenic, neurotrophic, and inflammatory signaling pathways, which are expected to contribute significantly to the therapeutic efficacy of diabetic wound healing.

Next, the bioactivities of *Saccharina japonica*‐derived Exos were evaluated by employing both human umbilical vein endothelial cells (HUVECs) and Schwann cells as model cells. First, the cytocompatibility of Exos was assessed using the cell counting kit‐8 (CCK‐8) assay. As shown in Figure 2h,i, the cell viability remained above 90% at varying concentrations (10–75 µg mL^−1^) of Exos after 24 h of co‐culture, demonstrating excellent cytocompatibility. Second, cellular uptake of Exos was assessed through co‐culture of 3,3′‐dioctadecyloxacarbocyanine perchlorate (DiO)‐labeled‐Exos with HUVECs or Schwann cells at 37 °C for 6 h, and then the intracellular fluorescence intensity was examined using confocal laser scanning microscopy (CLSM). As demonstrated in Figure 2j,k, pronounced green fluorescence intensities were observed in both DiO‐labeled‐Exos (50 µg mL^−1^)‐treated HUVECs and Schwann cells. Furthermore, the magnified images indicated that the DiO‐labeled‐Exos were predominantly localized around the cellular nuclei. Following this, the angiogenic potential of Exos was investigated in vitro by culturing HUVECs in complete DMEM medium supplemented with Exos. HUVECs cultured in complete DMEM medium were set as the blank group. The tube formation assay demonstrated that the addition of 50 µg mL^−1^ of Exos induced the formation of vascular‐like structures, characterized by increased tube length, number of junctions, and vascular coverage area after 6 h compared to the blank group (Figure 2l). Additionally, real‐time quantitative PCR (RT‐qPCR) analysis showed that the expression level of the key angiogenesis‐associated gene (vascular endothelial growth factor, VEGF) was significantly upregulated in HUVECs following a 6‐day co‐incubation with Exos (Figure 2m), confirming the pro‐angiogenic effects of Exos in vitro.

Furthermore, the neuroregenerative potential of Exos was assessed by culturing Schwann cells in complete DMEM medium supplemented with Exos for 6 days, with Schwann cells in complete DMEM medium serving as the blank group. Immunofluorescence staining revealed a significant increase in the fluorescence intensity of S100β, a Schwann cell marker, in the Exos‐treated group relative to the blank group, indicating that Exos effectively enhanced S100β expression in Schwann cells (Figure 2n). Moreover, mRNA expression levels of neurogenesis‐associated genes (brain‐derived neurotrophic factor (BDNF), S100β, and nerve growth factor (NGF)), as well as the protein level of BDNF, were significantly upregulated compared with the blank group (Figure 2o; Figure S4, Supporting Information). Collectively, these findings suggest that Exos possess both angiogenic and neurogenic bioactivities, demonstrating significant promise for neurovascular dual repair in diabetic wounds.

### 2.2 Preparation of Polyphenol‐Engineered Exosomes (CA@Exos) and Their Effectiveness in Combating AGEs Accumulation and Redox Imbalance

**Figure 3:**
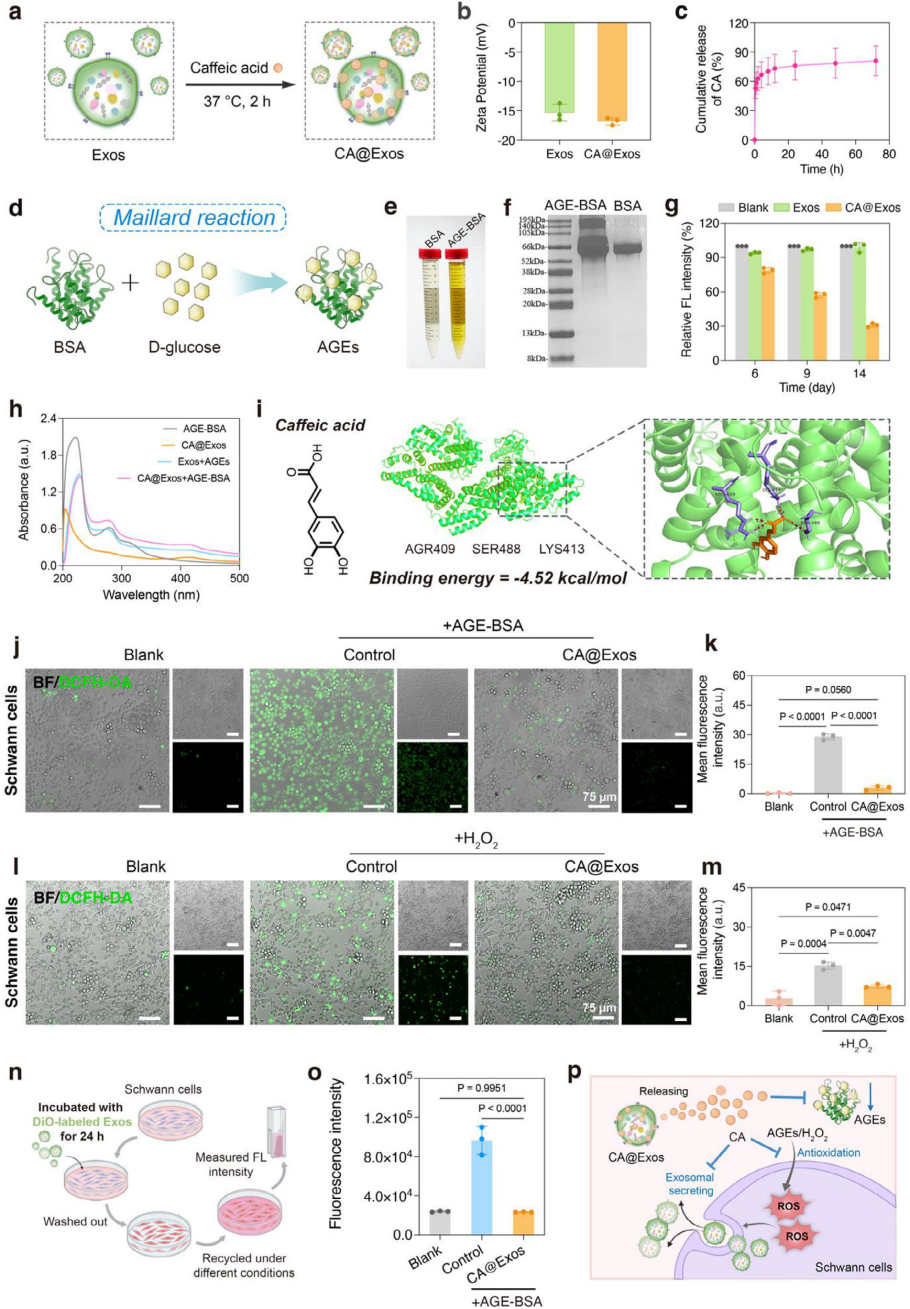
Characterization, antiglycation, and antioxidative evaluation of CA@Exos. a) Schematic illustration showing the preparation of CA@Exos. b) Zeta potential values of Exos and CA@Exos (*n* = 3). c) Release curve of CA from CA@Exos incubated in PBS (pH 7.4) containing 0.5% Tween 80 at 37 °C for 72 h (*n* = 3). d) Schematic illustration showing the synthesis of AGE‐BSA. e) Photograph showing the color of AGE‐BSA solution and BSA solution after incubation at 37 °C in the dark for 3 months. f) SDS‐PAGE electrophoresis showing the molecular weight of AGE‐BSA and BSA. g) Relative fluorescence intensity of mixed solution of BSA and D‐glucose with PBS, Exos, or CA@Exos at 6, 9, and 14 days. h) UV–vis spectra of CA@Exos solution, AGE‐BSA solution, and AGE‐BSA solution co‐cultured with Exos or CA@Exos (50 µg mL^−1^) for 3 days. i) The minimum energy conformation of CA‐AGEs complex obtained from molecular docking, showing surrounding amino acid residues and hydrogen bonds (yellow dashed lines) between CA and AGEs. j) Representative ROS staining images of Schwann cells under CA@Exos in the presence of AGE‐BSA. ROS was probed by DCFH‐DA (green). k) Quantification of mean fluorescence intensity in Schwann cells under different treatment conditions from CLSM images (*n* = 3). l) Representative ROS staining images of Schwann cells under CA@Exos in the presence of H_2_O_2_. ROS was probed by DCFH‐DA (green). m) Quantification of mean fluorescence intensity in Schwann cells under different treatment conditions from CLSM images (*n* = 3). n) Schematic illustration showing the procedures for the evaluation of DiO‐labeled‐Exos recycling. o) Fluorescence intensity (*λ*ex = 484 nm / *λ*em = 501 nm) of the culture medium. The Exos were labeled with DiO (*n* = 3). p) Schematic illustration showing the mechanism by which CA released from CA@Exos inhibits the formation of AGEs, mitigates oxidative stress, and prevents the oxidation‐induced exosomal secretion. Data are presented as mean values ± SD. Comparisons were performed by one‐way ANOVA followed by Tukey's multiple comparisons test in (k, m, o). Differences were considered statistically significant at *p* < 0.05.

AGEs were formed through the Maillard reaction between macromolecules (proteins, lipids, and nucleic acids) and reducing sugars.^[^ ^35^ ^]^ Previous studies reported that CA possessed the ability to inhibit the formation of AGEs through several mechanisms, including bonding to amino acid residues in proteins to block protein glycation sites, suppressing early glycation products, and trapping methylglyoxal (MGO, a precursor for AGEs formation).^[^ ^12^, ^36^ ^]^ However, the poor solubility of CA significantly limits its bioavailability. To circumvent this limitation, CA was loaded in *Saccharina japonica*‐derived Exos via a co‐incubation method at 37 °C for 2 h (**Figure** **3**a). The zeta potential values of Exos and CA‐loaded Exos (CA@Exos) were measured at −15.35 ± 1.44 mV and −16.82 ± 0.61 mV, respectively (Figure 3b), with the more negative charge in CA@Exos attributed to the incorporation of negatively charged groups (─COOH) from CA into Exos.^[^ ^37^ ^]^ Additionally, nano‐flow cytometry (Nano‐FCM) results further confirmed the presence of surface markers (CD9 and CD63) on both Exos and CA@Exos (Figure S5, Supporting Information), indicating that the loading of CA did not alter the surface markers of Exos. The encapsulating efficiency (EE) and loading efficiency (LE) of CA within CA@Exos were 10.78 ± 0.81% and 15.05 ± 0.96%, respectively (Figure S6, Supporting Information). Fourier‐transform infrared (FTIR) spectra of CA@Exos showed a characteristic absorption peak at 1620 cm^−1^, corresponding to the C═C stretching vibration of unsaturated acid side chain in CA^[^ ^38^ ^]^ (Figure S7, Supporting Information), further evidencing the successful loading of CA into CA@Exos. To assess the sustained release of CA from CA@Exos, the CA‐release curves were performed. As shown in Figure 3c and Figure S8, Supporting Information, the release of CA exhibited a sustained profile, with ≈81.06% released over 72 h in phosphate buffer saline (PBS, pH 7.4), suggesting that Exos served as effective nanocarriers for delivering CA in a sustained manner. Notably, the cumulative release of CA reached 96.09% ± 3.14% in Triton X‐100‐containing PBS over 72 h, significantly higher than that in PBS alone. This accelerated release in Triton X‐100‐containing PBS was triggered by exosomal membrane rupture, confirming that CA was predominantly encapsulated within the Exos. CA@Exos also demonstrated remarkable cytocompatibility, as evidenced by CCK‐8 assays performed on Schwann cells, mouse fibroblast cells (L929 cells), and HUVECs. After co‐culture with varying concentrations (10–75 µg mL^−1^) of CA@Exos, no cytotoxicity was observed even at a high concentration of 75 µg mL^−1^ (Figure S9, Supporting Information). These results laid the foundation for subsequent cellular experiments to further explore the biological functions of CA@Exos.

The inhibitory capacity of CA@Exos on the formation of AGEs was assessed in vitro. To simulate the conditions of AGE formation in the microenvironment of diabetic wounds, AGE‐bovine serum albumin (AGE‐BSA) was synthesized by incubating D‐glucose with BSA at 37 °C for 3 months (Figure 3d). As shown in Figure 3e, the color of AGE‐BSA solution was significantly darker than that of the pure BSA solution. Meanwhile, sodium dodecyl sulfate‐polyacrylamide gel electrophoresis (SDS‐PAGE) analysis revealed that the molecular weight of AGE‐BSA was higher than that of BSA (Figure 3f). The above results confirmed the successful synthesis of AGE‐BSA. To assess the inhibitory effect of CA@Exos on AGE‐BSA formation, the fluorescence intensity of the mixed solution of D‐glucose and BSA incubated with Exos or CA@Exos (50 µg mL^−1^) was measured, given that AGE‐BSA emits fluorescence upon excitation at 370 nm.^[^ ^39^ ^]^ The results revealed that the relative fluorescence intensity of the solution in the presence of CA@Exos was significantly lower than that of the solution without CA@Exos (blank) over a duration of 14 days (Figure 3g), indicating that CA@Exos effectively inhibited AGE formation. In contrast, the relative fluorescence intensity of the solution in the presence of Exos remained consistently above 94% over a duration of 14 days, suggesting negligible antiglycative activity. These findings confirm that the inhibitory effects of CA@Exos on AGE formation were predominantly attributed to the encapsulated CA, rather than the Exos themselves. Furthermore, the binding affinity of CA released from CA@Exos toward preformed AGE‐BSA was assessed. As shown in Figure 3h, the UV–vis absorption peak at 278 nm exhibited a marked increase in the AGE‐BSA solution after incubation with CA@Exos for 3 days, whereas no significant change was observed with Exos alone. This spectral shift confirmed that the interaction was attributed to CA rather than the Exos themselves. To further investigate the interactions between CA and AGEs, molecular docking analysis was performed. The spatial conformation and key amino acid residues involved in the interaction between CA and AGEs were illustrated in Figure 3i. The results revealed that CA exhibited stable binding (Δ*G* = −4.52 kcal mol^−1^) with AGEs, forming hydrogen bonds with LYS413, ARG409, and SER488. The binding to glycation‐prone residues (Arg/Lys) of AGEs suggested that CA can interfere with the AGEs‐RAGE interaction by occupying critical binding sites on AGE‐BSA.^[^ ^40^, ^41^ ^]^

Previous studies have shown that the accumulation of AGEs can activate RAGE, resulting in elevated intracellular ROS production.^[^ ^42^ ^]^ To evaluate the protective effect of CA@Exos against AGE‐BSA‐induced oxidative damage, Schwann cells were co‐cultured with AGE‐BSA and CA@Exos for 6 h, followed by ROS detection using 2′,7′‐dichlorodihydrofluorescein diacetate (DCFH‐DA) probe. CLSM images revealed a remarkable reduction in the intensity of green fluorescence in Schwann cells treated with 50 µg mL^−1^ CA@Exos, indicating decreased ROS (Figure 3j,k). This reduction may be attributed to the interaction between CA and AGE‐BSA, leading to the formation of complexes that impede AGE‐BSA binding to the RAGE, thereby inhibiting the production of intracellular ROS.

CA@Exos also exhibited potent antioxidant properties capable of alleviating oxidative stress in the diabetic wound microenvironment. This was demonstrated by treating Schwann cells with 50 µm H_2_O_2_ and CA@Exos, followed by detection of intracellular ROS levels using the DCFH‐DA fluorescence probe. As shown in Figure 3l,m, the intracellular green fluorescence intensity was significantly reduced in CA@Exos‐treated group compared to the control group. Flow cytometry analysis further revealed that the proportion of Schwann cells exhibiting excessive ROS decreased from 27.03% ± 2.01% to 15.00% ± 1.28% in the CA@Exos‐treated groups (Figure S10, Supporting Information). These findings indicate that CA@Exos can effectively mitigate oxidative stress induced by both elevated AGEs and ROS within the microenvironments of diabetic wounds.

Oxidative stress is known to impair the endocytic cycle and activate exocytosis, thereby reducing the bioavailability of nanomedicines within cells.^[^ ^43^ ^]^ To assess the role of CA@Exos in alleviating oxidative stress and modulating exosomal secretion dynamics, Schwann cells were pre‐incubated with DiO‐labeled‐Exos (50 µg mL^−1^) for 24 h to establish a baseline exosomal uptake model. Subsequently, cells were divided into three experimental groups: 1) **CA@Exos + AGE‐BSA (200 µg mL^−1^)**, 2) **AGE‐BSA alone** (oxidative stress control), and 3) **untreated cells** (baseline control). Following 12 h of co‐culture, extracellular DiO fluorescence was quantified via fluorescence spectrometer (*λ*ex/em = 484/501 nm), which revealed that CA@Exos treatment significantly **reduced exosomal** secretion compared to the AGE‐BSA group (fluorescence intensity: 23293 ± 304 a.u. versus 96586 ± 14270 a.u.), restoring secretion levels to near‐baseline conditions (versus untreated: 23931±  669 a.u.) (Figure 3n,o). This result indicated that CA@Exos alleviated the AGE‐BSA‐induced oxidative damage in Schwann cells, thereby reducing the secretion of DiO‐labeled‐Exos by Schwann cells, which potentially enhanced exosomal bioavailability. Collectively, these results indicate that CA released from CA@Exos not only inhibits AGE formation but also interacts with preformed AGEs, forming a complex that may disrupt AGEs‐RAGE binding. This dual mechanism effectively mitigates oxidative stress‐induced cellular damage, highlighting the potential of CA in AGE‐related pathologies (Figure 3p).

### 2.3 CA@Exos Promote Macrophage Reprogramming, Fibroblast Migration, and Reconstruction of Neurovascular Networks in vitro

**Figure 4:**
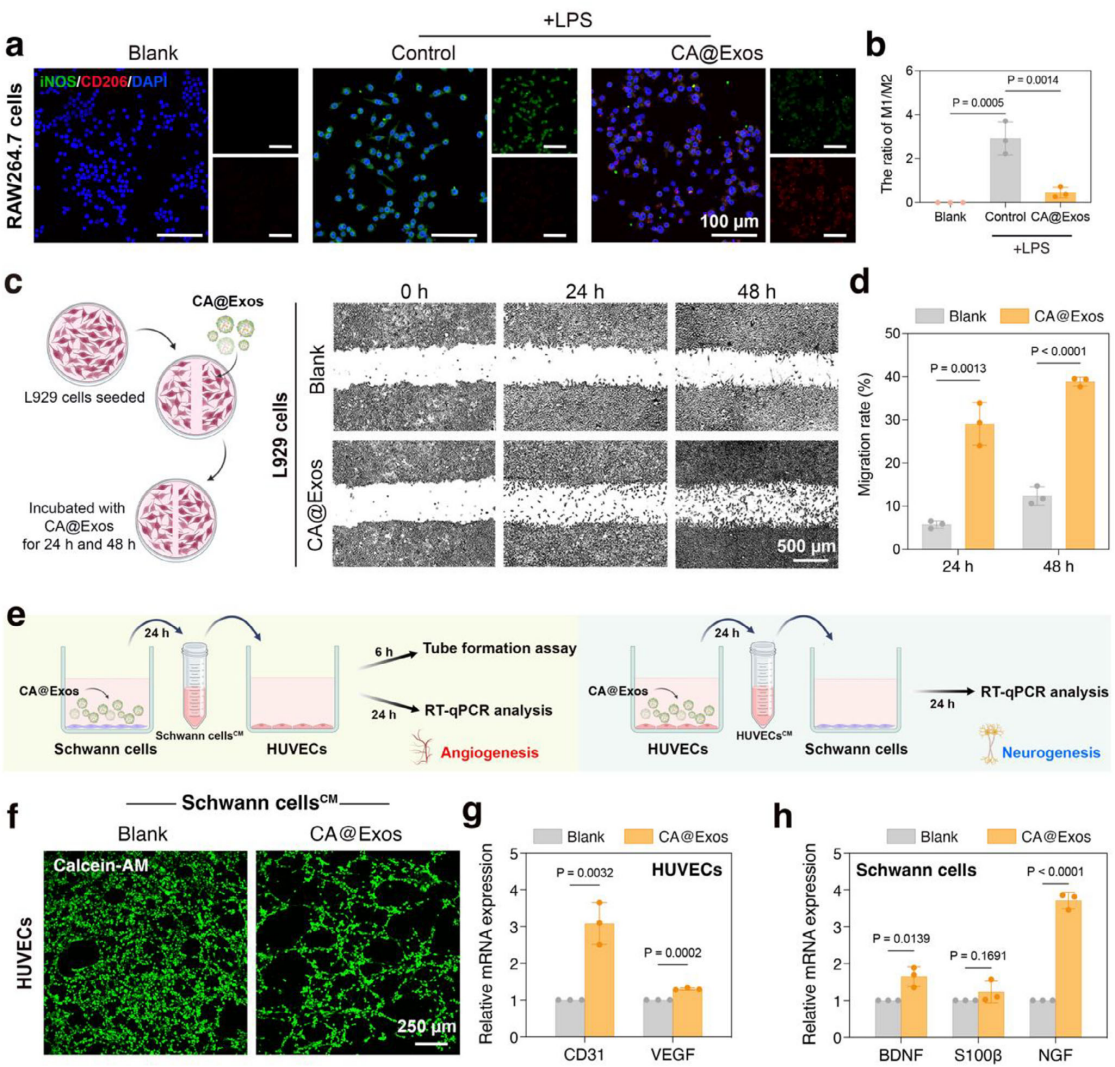
The effects of CA@Exos on anti‐inflammation, cellular migration, and neurovascular network crosstalk. a) Immunofluorescence images of CD68 (green) and CD206 (red) staining in RAW264.7 cells cultured with CA@Exos for 24 h. b) Quantification of the ratio of M1/M2 (*n* = 3) c) Representative images of scratch assay of L929 cells at 24 and 48 h. The pseudo color was added by image J software. d) Quantification of cell migration rate at 24 and 48 h (*n* = 3). e) Schematic illustration showing the design of indirect co‐culture system to investigate the neurovascular crosstalk. Left panel showing the effect of Schwann cells^CM^ on angiogenesis in HUVECs. Right panel showing the effect of HUVECs^CM^ on neurogenesis in Schwann cells. f) Fluorescence images of Matrigel tube formation assay. HUVECs were cultured in DMEM (Blank) and Schwann cells^CM^ for 6 h. g) RT‐qPCR analysis of the expression levels of CD31 and VEGF in HUVECs incubated with Schwann cells^CM^ for 24 h (*n* = 3). h) RT‐qPCR analysis of the expression levels of BDNF, S100β, and NGF in Schwann cells incubated with HUVECs^CM^ for 24 h (*n* = 3). Data are presented as mean values ± SD. Comparisons were performed by one‐way ANOVA followed by Tukey's multiple comparisons test in (b) and unpaired two‐tailed Student's *t* test in (d, g, h). Differences were considered statistically significant at *p* < 0.05.

Modulating macrophage polarization is a crucial process in the healing of diabetic wounds. During the inflammatory phase, M1 macrophages predominantly serve an antibacterial function. However, excessive infiltration of M1 macrophages can lead to the secretion of pro‐inflammatory cytokines, which prolong the inflammatory stage, ultimately hindering the wound healing process.^[^ ^44^ ^]^ Therefore, the timely conversion of the macrophage phenotypes from M1 to M2 is essential for transitioning the inflammatory phase into a proliferative phase, thus expediting wound healing.^[^ ^45^ ^]^ To explore the effects of CA@Exos on macrophage polarization, mouse leukemia cells of monocyte macrophage (RAW264.7) cells were stimulated with lipopolysaccharide (LPS) for 24 h to induce M1 polarization. Subsequently, these cells were cultured in complete DMEM medium supplemented with CA@Exos (50 µg mL^−1^) for 24 h. Immunofluorescence staining images of RAW264.7 cells demonstrated that CA@Exos‐treated cells exhibited diminished fluorescence signals (green) for inducible nitric oxide synthase (iNOS) and heightened fluorescence signals (red) for mannose receptor (CD206) compared to LPS‐treated cells (**Figure** **4**a). Quantitative analysis further demonstrated that the M1/M2 ratio in CA@Exos‐treated cells (0.45% ± 0.24%) was lower than that in LPS‐treated cells (2.92% ± 0.75%) (Figure 4b). These results reveal that CA@Exos effectively regulate macrophage polarization, favoring an anti‐inflammatory and pro‐healing M2 phenotype that is critical for restoring immune homeostasis and enhancing tissue repair in diabetic wounds.

Besides, the migration of fibroblasts is pivotal in the wound healing process, as they gradually enhance collagen deposition, thereby forming a stable extracellular matrix that supports new tissue formation.^[^ ^24^ ^]^ In this aspect, the ability of CA@Exos to promote cell migration was further assessed by a scratch test. As shown in Figure 4c, L929 cells in the CA@Exos‐treated group exhibited a pronounced tendency to migrate toward and populate the wound site over 48 h, whereas cells in the blank group remained randomly distributed along the scratch margin. The migration ratio of L929 cells treated with CA@Exos was 29.09% ± 4.95% at 24 h and increased to 38.88% ± 1.03% at 48 h (Figure 4d), significantly higher than that of the blank group, indicating a sustained pro‐migratory effect of CA@Exos. These results suggest that CA@Exos exhibit a potent ability to enhance fibroblast migration, thereby accelerating re‐epithelialization in diabetic wound healing.

The reconstruction of neurovascular network plays a crucial role in diabetic wound healing.^[^ ^23^, ^46^ ^]^ Previous studies have highlighted the critical role of angiogenesis‐related factors in supporting neuron survival and facilitating axonal growth.^[^ ^47^ ^]^ Conversely, neurotrophic‐related factors also promoted angiogenesis, highlighting the intricate crosstalk between neural and vascular regeneration. To explore the potential effects of the secretory microenvironment on cellular behavior and functionality, the crosstalk between HUVECs and Schwann cells in the presence of CA@Exos was evaluated using an indirect co‐culture system (Figure 4e). First, the conditioned medium (CM) from the Schwann cells‐CA@Exos co‐culture system (Schwann cells^CM^) was collected to assess its effects on the behaviors of HUVECs. Notably, over 93% of DiO‐labeled CA@Exos were internalized by Schwann cells (Figure S11, Supporting Information), indicating efficient uptake of CA@Exos by the cells. Compared to the blank group, HUVECs cultured in Schwann cells^CM^ supplemented with CA@Exos exhibited enhanced tubular structure formation, indicating its remarkable ability to enhance vascularization (Figure 4f). Furthermore, RT‐qPCR analysis showed that the expression levels of platelet endothelial cell adhesion molecule‐1 (CD31) and VEGF genes in HUVECs were significantly upregulated when co‐cultured with Schwann cells^CM^ supplemented with CA@Exos (Figure 4g), further corroborating the role of CA@Exos in promoting vascularization. Second, to evaluate whether angiogenesis reciprocally influences neurogenesis, the expression levels of neurogenic markers, such as BDNF, S100β, and NGF, were evaluated using RT‐qPCR analysis. As shown in Figure 4h, co‐culture of Schwann cells with HUVECs^CM^ supplemented with CA@Exos led to a significantly upregulated expression of these neurogenic genes compared to the blank group, indicating a pro‐neurogenic effect of CA@Exos by stimulating angiogenesis. These results indicate that the secretory microenvironment generated after cellular uptake of CA@Exos promotes the crosstalk between Schwann cells and HUVECs. By fostering this bidirectional communication, CA@Exos regulates the reconstruction of the neurovascular network, thereby facilitating effective tissue regeneration during diabetic wound healing.

### 2.4 Preparation and Characterization of Electroconductive Microneedles Loaded With CA@Exos

**Figure 5:**
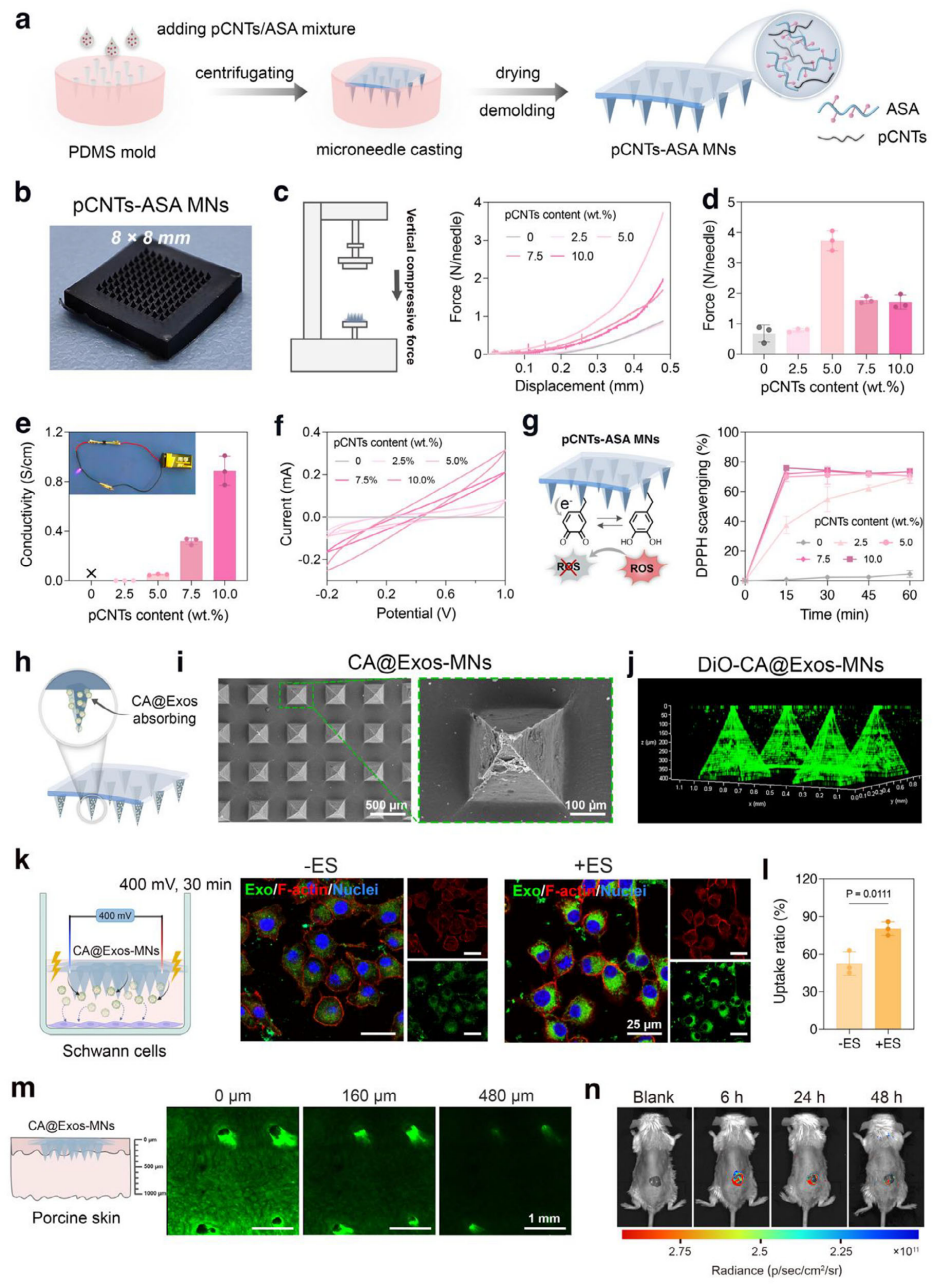
Preparation and characterization CA@Exos‐loaded electroconductive MNs (CA@Exos‐MNs). a) Schematic illustration showing the fabrication process of pCNTs‐ASA MNs. b) The macroscopic image of pCNTs‐ASA MNs. c) The compressive force‐displacement curves of pCNTs‐ASA MNs with varying content of pCNTs. d) The compressive force (N per needle) of pCNTs‐ASA MNs (*n* = 3). e) Conductivity and f) cyclic voltammetry (CV) curves of pCNTs‐ASA MNs with varying content of pCNTs. The insert shows the pCNTs‐ASA MNs‐integrated circuit lighted up an LED. g) DPPH scavenging efficacy of pCNTs‐ASA MNs with varying content of pCNTs (*n* = 3). h) Schematic illustration showing the preparation of the CA@Exos‐MNs by physically absorbing CA@Exos onto the pCNTs‐ASA MNs. i) The SEM images of CA@Exos‐MNs. j) Representative 3D CLSM image of DiO‐labeled‐CA@Exos‐MNs. k) Schematic illustration showing the experimental setup for applying ES (400 mV, 30 min) to Schwann cells cultured below DiO‐labeled‐CA@Exos‐MNs. CLSM images of the cellular uptake of DiO‐labeled‐CA@Exos in Schwann cells with or without ES. The cell nuclei were stained using DAPI (blue), the F‐actin was stained using phalloidin (red), and Exos were labeled with DiO (green). l) The uptake ratio of CA@Exos in Schwann cells with or without ES (*n* = 3). m) CLSM images of DiO‐labeled‐CA@Exos released from MNs and penetrating to porcine skins at different depths. n) In vivo fluorescence images of mice after application of DiD‐labeled‐CA@Exos‐MNs at wound on the back for different periods (6, 24, and 48 h). Data are presented as mean values ± SD. Comparisons were performed by unpaired two‐tailed Student's t test in (l). Differences were considered statistically significant at *p* < 0.05.

To enable efficient delivery of CA@Exos into wound tissues, electroconductive microneedles were designed. The matrix of MNs was made from octenyl succinic anhydride‐grafted agarose (ASA), which was synthesized through the functionalization of agarose with octenyl succinic anhydride (OSA) via esterification reaction (Figure S12, Supporting Information). To endow the MNs with conductivity, polydopamine‐decorated CNTs (pCNTs) (Figure S13, Supporting Information) were additionally introduced into the OSA network. To fabricate pCNTs‐incorporated ASA microneedles (pCNTs‐ASA MNs), pCNTs and ASA were mixed in DMSO, and the resulting mixture was cast into a PDMS mold, followed by centrifugation (**Figure** **5**a). After drying at 60 °C in an oven for 8 h, the pCNTs‐ASA MNs array was fabricated, yielding an 8 × 8 mm^2^ patch containing 100 vertically aligned needles (Figure 5b).

To optimize the composition of MNs for loading CA@Exos, a series of pCNTs‐ASA MNs with varying pCNTs content (0, 2.5, 5, 7.5, and 10 wt.%) were fabricated, and their mechanical and conductive properties of MNs were systematically evaluated. Compressive tests showed that the mechanical strength of the MNs was enhanced with the increased pCNTs content due to the nanoreinforcing effect of pCNTs (Figure 5c,d). However, when the pCNTs content was higher than 5 wt.%, the mechanical strength of the MNs decreased, likely due to structural inhomogeneity or brittleness induced by excessive pCNTs. At 5 wt.% of pCNTs, the MNs displayed a maximum of mechanical strength (3.73 ± 0.33 N per needle), sufficient for effective skin penetration.^[^ ^48^ ^]^ To evaluate conductivity, square pCNTs‐ASA films (10 × 10 mm) with a thickness of 0.6 mm were fabricated (see the details in Supporting Information), and the electroconductivity of various films was examined using a four‐point probe method. As shown in Figure 5e, the conductivity of pCNTs‐ASA films was increased with the increase of pCNTs content. In addition, the cyclic voltammetry (CV) measurements demonstrated that the pCNTs‐ASA films showed enhanced oxidation and reduction currents compared with the ASA films (Figure 5f), which indicated improved electrochemical activity and conductivity due to the enhanced charge transfer properties from the pCNTs. Furthermore, the antioxidant capacity of pCNTs‐ASA MNs was verified by the 2,2‐diphenyl‐1‐picrylhydrazyl (DPPH) free radical scavenging test. As shown in Figure 5g, after the addition of pCNTs, the DPPH scavenging ratio of the MNs was significantly increased and reached about 70.67% at 5 wt.% of pCNTs, which was comparable to the value obtained with 10 wt.% pCNTs (about 73.47%). The antioxidant effect of the pCNTs‐ASA MNs originated from the inherent scavenging ability of catechol groups on pCNTs.^[^ ^49^ ^]^ Taken together, the pCNTs‐ASA MNs with 5 wt.% of pCNTs content, exhibiting a mechanical strength of 3.73 ± 0.33 N per needle, conductivity of 0.049 ± 0.004 S cm^−1^, and DPPH scavenging efficiency of 70.66% ± 5.07%, are suitable for penetrating the human dermal barrier to deliver the CA@Exos.

Next, CA@Exos were physically absorbed onto the pCNTs‐ASA MNs, resulting in the formation of CA@Exos‐MNs (Figure 5h). As shown in Figure S14 (Supporting Information), the adsorption of CA@Exos did not significantly alter the macroscopic morphology or surface topography of the microneedle array. However, scanning electron microscope (SEM) imaging revealed increased surface roughness at the microneedle tips in the CA@Exos‐MNs compared to bare MNs (Figure 5i and Figure S15, Supporting Information), directly attributable to the integration of CA@Exos. The loading amount and encapsulation efficiency (EE) of CA@Exos in each CA@Exos‐MNs were calculated to be 31.67 ± 2.85 µg and 15.84% ± 1.42%, respectively (Figure S16, Supporting Information). Additionally, the 3D reconstructed fluorescence image also demonstrated a uniform and spatially coherent distribution of DiO‐labeled‐CA@Exos at the tips of the MNs (Figure 5j). Notably, compression testing showed that CA@Exos‐MNs retained structural integrity comparable to unmodified MNs, with a mean fracture force of 3.35 ± 0.17 N per needle (Figure S17a,b, Supporting Information), confirming their mechanical robustness. Additionally, CA@Exos‐MNs maintained favorable electrical conductivity (0.059 ± 0.002 S cm^−1^; Figure S17c, Supporting Information), ensuring effective electrical signal transmission. These results collectively confirm that CA@Exos functionalization preserves both the structural durability and electrical functionality required for In vivo therapeutic applications. The biocompatibility of CA@Exos‐MNs was assessed through Live/Dead staining and CCK‐8 assays, both of which confirmed the cytocompatibility of CA@Exos‐MNs in Schwann cells (Figure S18, Supporting Information), thereby ensuring their biosafety for potential In vivo applications.

Previous studies have indicated that ES can promote the cellular uptake of the nanomedicines.^[^ ^50^ ^]^ To investigate the cellular uptake of CA@Exos from MNs under ES, DiO‐labeled‐CA@Exos‐MNs were gently placed on the Schwann cells and incubated for 1.5 h, followed by the application of ES (400 mV) for 0.5 h. Then the cells were stained with Phalloidin and DAPI to observe the internalization of DiO‐labeled‐CA@Exos in Schwann cells under CLSM. As shown in Figure 5k, the green fluorescence was primarily located in the cytoplasm of Schwann cells, with significantly stronger intensities in the ES group compared to the non‐ES group. Quantitative analysis exhibited approximately a 1.53‐fold increase in cellular uptake ratio of CA@Exos following ES compared to cells without ES (Figure 5l), confirming that ES significantly enhanced CA@Exos internalization. In addition, electrically stimulated cells demonstrated viability exceeding control levels, confirming the biocompatibility and safety profile of the 400 mV stimulation parameter (Figure S19, Supporting Information). These results indicate that the ES enhances the cellular uptake of CA@Exos, potentially augmenting the bioavailability and amplifying multifunctional biological effects of CA@Exos.

Meanwhile, the pCNTs‐ASA MNs facilitated an efficient release of CA@Exos, with 75.26% ± 8.60% released over 48 h in PBS (Figure S20, Supporting Information). To evaluate skin penetration, DiO‐labeled CA@Exos‐MNs were applied to porcine skin for 20 min. CLSM images revealed that the microchannels generated by the insertion of MNs, along with the fluorescence signals of DiO‐labeled‐CA@Exos, reached a depth of 480 µm in porcine skin (Figure 5m), indicating that the conductive MNs exhibited excellent penetration, thereby enabling the effective and deep delivery of CA@Exos into the skin tissue. Subsequently, the in vivo retention of CA@Exos was assessed by applying the DiD‐labeled‐CA@Exos‐MNs on the dorsal skin wounds of BALB/c mice and monitored using an in vivo imaging system. The fluorescence signal emanating from DiD‐labeled‐CA@Exos was prominently detected at 6 h and persisted for up to 48 h (Figure 5n), indicating that the MNs promoted sustained release of CA@Exos in vivo. This observation holds significant implications for determining the optimal replacement interval for CA@Exos‐MNs in future in vivo investigations. In summary, the conductive pCNTs‐ASA MNs exemplified outstanding biocompatibility and the ability to load CA@Exos for subsequent transdermal delivery and internalization in conjunction with electrical stimulation.

### 2.5 In vivo Efficacy of CA@Exos‐MNs Combined With ES on Diabetic Wound Healing

**Figure 6:**
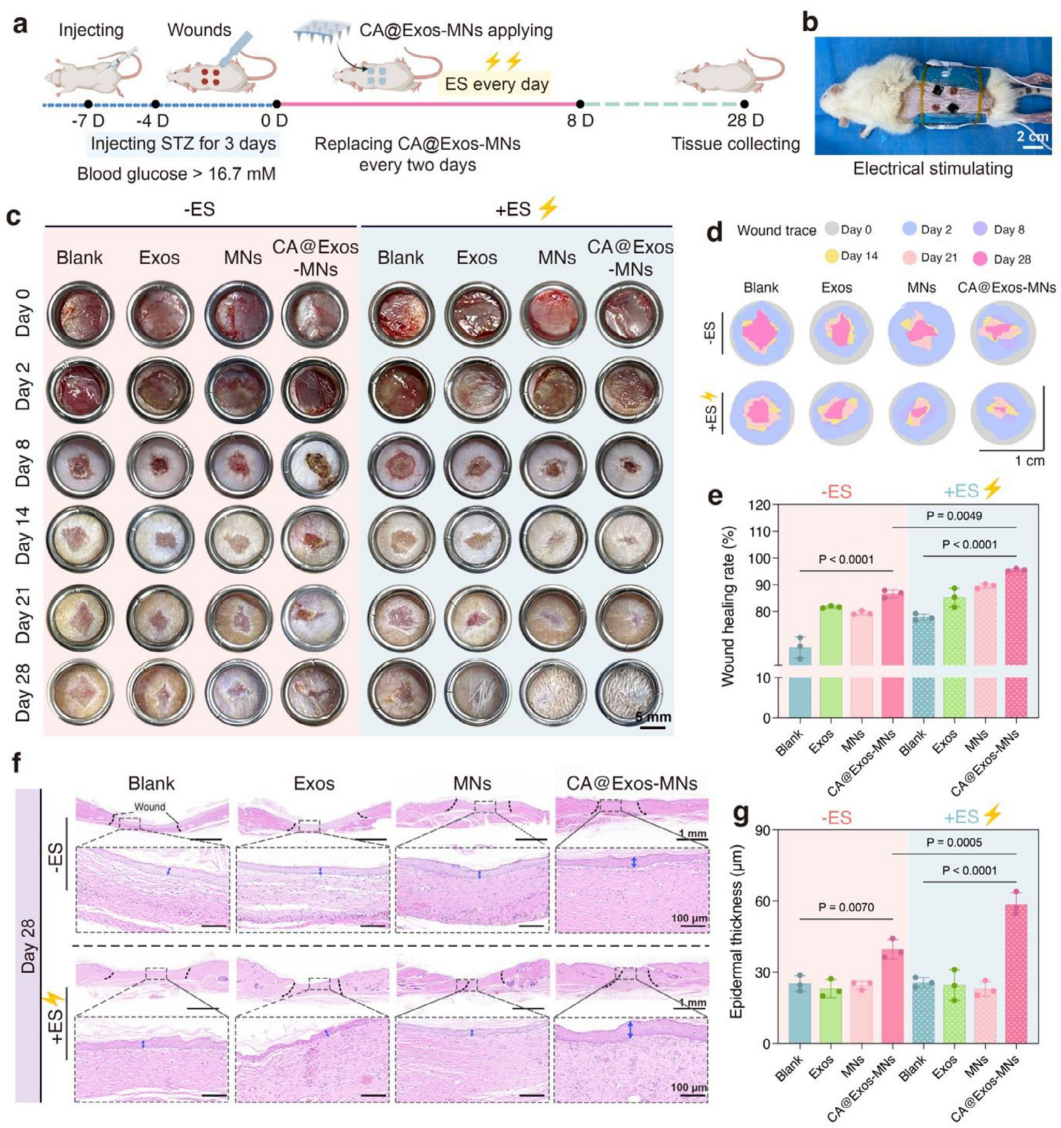
In vivo therapeutic efficacy of CA@Exos‐MNs combined with ES in diabetic wound healing. a) Schematic illustration of treatment schedule for the diabetic full‐thickness skin wound in SD rats. b) Photograph showing the treatment of CA@Exos‐MNs combined with ES to SD rat. c) Photographs of wounds and d) traces of wound closure in different treatment groups from day 0 to day 28. e) Quantification of wound healing rate on day 28 (*n* = 3). f) Representative images of H&E staining of tissue sections in different treatment groups on day 28. Black dotted lines indicate wound margins, and the blue double arrows indicate the epidermal thickness. The inset gray dotted boxes highlight the areas of interest. g) Quantification of epidermal thickness of the wounded skin on day 28 (*n* = 3). Data are presented as mean values ± SD. Comparisons were performed by one‐way ANOVA followed by Tukey's multiple comparisons test in (e, g). Differences were considered statistically significant at *p* < 0.05.

To investigate the in vivo efficacy of CA@Exos‐MNs combined with ES on diabetic wound healing, a streptozotocin (STZ)‐induced diabetic rat model with full‐thickness skin wounds was established (**Figure** **6**a). The successful induction of diabetes was validated by monitoring the body weight and blood glucose levels of Sprague‐Dawley (SD) rats in the PBS‐ and STZ‐injected groups over time. The results showed that the STZ‐induced diabetic rats exhibited significant weight loss, higher blood glucose level, and polyuria/polydipsia compared with PBS‐treated rats (Figure S21, Supporting Information). The diabetic rats were randomly divided into 8 treatment groups, including 1) Blank group (without any treatment), 2) Exos group, 3) MNs group, 4) CA@Exos‐MNs group, 5) ES group, 6) Exos + ES group, 7) MNs + ES group, and 8) CA@Exos‐MNs + ES group. Exos were administered topically (31 µL of 1 mg mL^−1^ solution, matching the CA@Exos amount delivered via CA@Exos‐MNs). For ES groups, ES was applied using an ES device for 60 min per day during the initial 8 days post‐implantation (Figure 6b). The wound changes were photographed on days 2, 8, 14, 21, and 28 (Figure 6c). Macroscopically, the wound area in CA@Exos‐MNs‐treated group was smaller compared to those in the Exos‐treated, MNs‐treated, and Blank groups by the end of the observation period. In the ES‐treated groups, wounds exhibited a reduction in area, accompanied by enhanced neogenesis of hair follicles, when compared to the corresponding non‐ES groups. Notably, the CA@Exos‐MNs + ES group demonstrated the most rapid reduction in wound area among all groups throughout the healing process. Wound traces were marked with different colors according to the photographs during the wound healing (Figure 6d). The wound healing rate in CA@Exos‐MNs groups (86.65% ± 1.47%) exhibited significantly greater compared to the Exos groups (81.75% ± 0.44%), indicating that MNs‐mediated delivery of CA@Exos enhanced wound healing rate more effectively than Exos administration alone (Figure 6e). Additionally, the wound healing rate in CA@Exos‐MNs + ES group (95.61% ± 0.49%) was faster than that in Exos + ES group (85.26% ± 3.52%), representing an ≈1.12‐fold increase. This enhancement was more pronounced compared to the difference between CA@Exos‐MNs and Exos groups without ES (1.06‐fold). These results suggest that ES facilitated the internalization of CA@Exos by tissues to exert biological activity, thereby accelerating the wound healing process. Furthermore, the wound healing rate in ES‐treated groups was significantly higher than that in the non‐ES groups, highlighting the beneficial role of ES in wound recovery. Among all treatment groups, the wounds in the CA@Exos‐MNs + ES group exhibited the most rapid and pronounced healing rate. These results demonstrate that CA@Exos‐MNs combined with ES significantly enhance diabetic wound healing. The penetration ability of MNs facilitated the transdermal and sustained delivery of CA@Exos, as CA‐only groups demonstrated minimal efficacy in wound size reduction compared to the Blank group (Figure S22, Supporting Information). Meanwhile, ES not only promoted cellular uptake of CA@Exos but also further promoted wound healing by establishing an electrical field through the conductive MNs. This synergistic approach accelerated wound healing and regeneration of hair follicle, highlighting its potential as an effective strategy for diabetic wound management.

Hematoxylin and eosin (H&E) staining was conducted to evaluate the quality of the regenerated skin wound tissues on day 28. The H&E staining images revealed that the wound margin exhibited the widest gap in the Blank group and the most narrowed in the CA@Exos‐MNs + ES group, in comparison to the other groups (Figure 6f). Both the MNs + ES and CA@Exos‐MNs + ES groups exhibited more pronounced wound closure compared to the groups without ES. Notably, newly formed hair follicles were observed in these two groups, indicating active tissue regeneration. These results suggest that conductive MNs could transmit the electrical signals to the wound site, thereby promoting tissue repair and facilitating the regeneration of hair follicles. Quantitative analysis further indicated that the CA@Exos‐MNs + ES group exhibited the greatest epidermal thickness compared to the other groups (Figure 6g), signifying an enhanced capability for tissue regeneration facilitated by the combination of CA@Exos‐MNs and ES during the wound healing process.

**Figure 7:**
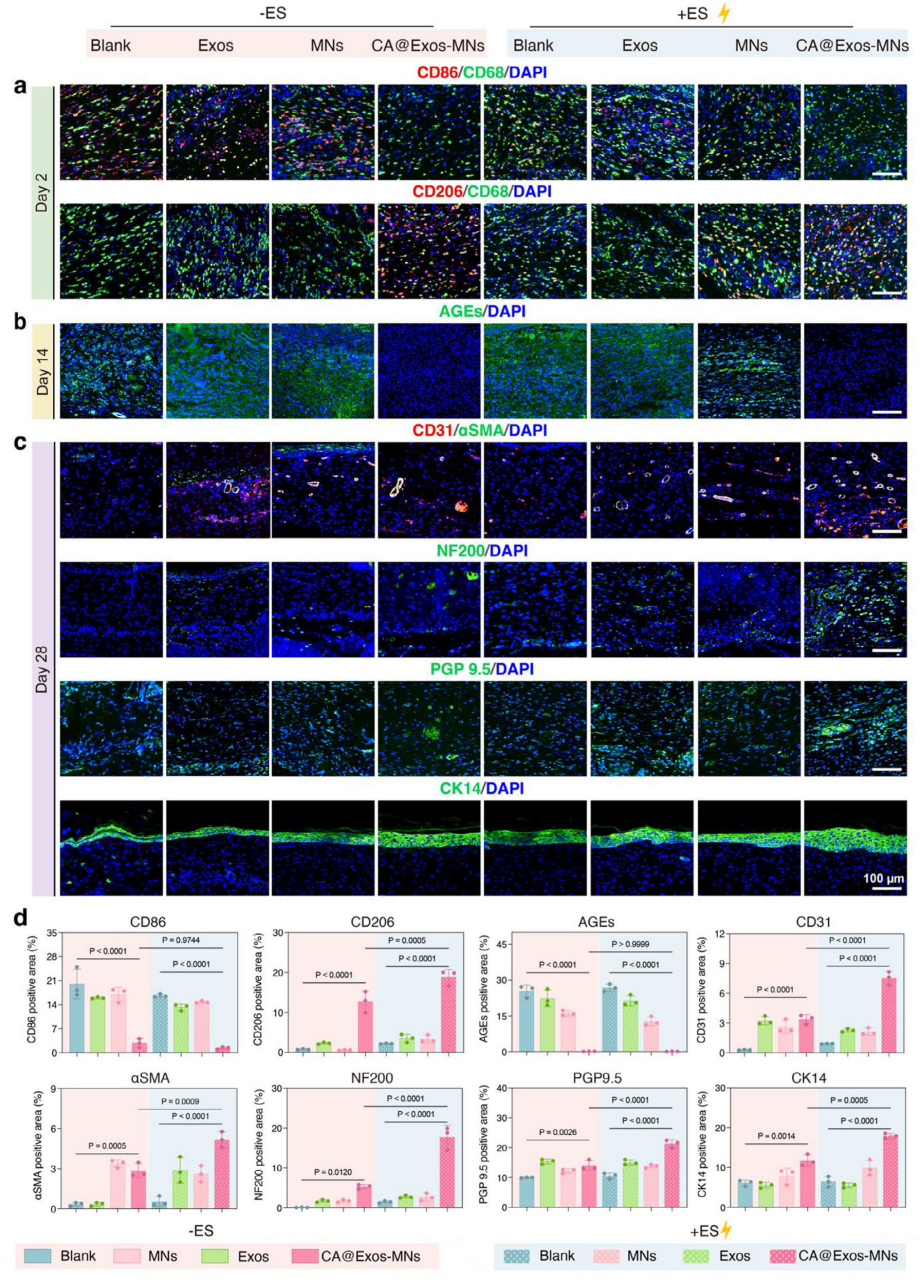
Regulation of inflammation, inhibition of glycation, and promotion of neurovascular network reconstruction by CA@Exos‐MNs combined with ES in diabetic wound healing. a) Immunofluorescence staining for the pan macrophage marker CD68 (green) and co‐stained for either CD86 (red) or CD206 (red) in tissue sections obtained from different treatment groups on day 2. b) Immunofluorescence staining for AGEs (green) in tissue sections obtained from different treatment groups on day 14. c) Immunofluorescence staining for CD31 (red)/αSMA (green), NF200 (green), PGP 9.5 (green), and CK14 (green) in tissue sections obtained from different treatment groups on day 28. d) Quantification of CD86, CD206, AGEs, CD31, αSMA, NF200, PGP 9.5, and CK14 positive area in tissue sections from immunofluorescence staining images (*n* = 3). Data are presented as mean values ± SD. Comparisons were performed by one‐way ANOVA followed by Tukey's multiple comparisons test in (d). Differences were considered statistically significant at *p* < 0.05.

Inflammation and immune response also play crucial roles in wound healing. However, the excessive or prolonged activation of inflammation and immune response impairs the healing process.^[^ ^51^ ^]^ To investigate the potential of CA@Exos‐MNs + ES in modulating macrophage polarization and attenuating inflammation, H&E staining was performed on skin wound tissues harvested on day 2. As shown in Figure S23 (Supporting Information), prominent immune cell infiltration was observed in the Blank group, whereas inflammation was significantly alleviated in both the CA@Exos‐MNs and CA@Exos‐MNs + ES groups. Furthermore, immunofluorescence staining for CD68 (macrophage biomarker), CD86 (M1 macrophage biomarker), and CD206 (M2 macrophage biomarker) was also performed on the tissue sections from wounds on day 2 (**Figure** **7**a). In comparison to the other groups, an increased expression of CD206 alongside a reduced expression of CD86 was observed in both the CA@Exos‐MNs and CA@Exos‐MNs + ES groups, suggesting that CA@Exos‐MNs effectively promoted a transition from an M1 macrophage‐dominant environment to one enriched with M2 macrophages. Moreover, the CA@Exos‐MNs + ES group showed a more pronounced M2 polarization than CA@Exos‐MNs group, suggesting that ES further enhanced macrophage reprogramming. The above results demonstrate that CA@Exos‐MNs, particularly in combination with ES, effectively suppress the early inflammation by promoting M2 macrophage polarization, thereby facilitating the transition from the inflammatory to the proliferative phase of wound healing and accelerating diabetic wound repair.

To further assess the ability of CA@Exos to inhibit the formation of AGEs, immunofluorescence staining of AGEs was performed on skin wound tissues harvested at day 14. As shown in Figure 7b, the expression of AGEs was significantly diminished in both CA@Exos‐MNs and CA@Exos‐MNs + ES groups, indicating the in vivo inhibitory effect of CA@Exos on AGE formation. The decreased formation of AGEs likely contributed to the reduction of ROS generation, thereby alleviating diabetic neuropathy and angiopathy.

To further assess the maturation of the newly regenerated tissues, skin wound tissues at day 28 were harvested for immunofluorescence analysis (Figure 7c). Efficient vascularization is critical for the adequate delivery of oxygen and nutrients, which are essential for facilitating wound healing.^[^ ^52^ ^]^ CD31 serves as a specific endothelial cell marker, while α‐smooth muscle actin (αSMA) identifies mature vascular smooth muscle cells and activated myofibroblasts, enabling discrimination of vascular maturity through co‐localization analysis,^[^ ^53^ ^]^ thus dual immunofluorescence co‐staining of CD31 and αSMA was performed on wound sections to observe angiogenesis In vivo. As shown in Figure 7c, the CA@Exos‐MNs + ES group demonstrated significantly increased CD31/α‐SMA co‐localization in comparison to other groups, indicating enhanced neovascularization and maturation of blood vessels in diabetic wounds. These results suggest that CA@Exos‐MNs + ES promotes both endothelial sprouting (CD31⁺) and functional vessel formation (α‐SMA⁺), which is essential for effective diabetic wound healing. Moreover, nerve fibers, integral components of skin tissue found within the epidermis and dermis layers, play a pivotal role in mediating wound healing.^[^ ^54^ ^]^ Immunofluorescence staining revealed that the expression of neurofilament 200 (NF200) was significantly elevated in the CA@Exos‐MNs + ES group compared to the other groups, suggesting that CA@Exos‐MNs + ES enhanced neuroregeneration at the wound sites. Additionally, immunofluorescence staining for protein gene product 9.5 (PGP 9.5), a neuron‐specific protein present in neurons and nerve fibers,^[^ ^55^ ^]^ also revealed a notable increase in nerve density in the CA@Exos‐MNs + ES group. These findings suggest that CA@Exos‐MNs combined with ES effectively promote angiogenesis and neural regeneration, thereby supporting tissue repair and functional recovery at the wound site.

The epidermis is a complex and multilayered squamous epithelium, where the formation of functional keratin, such as cytokeratin 14 (CK14), is essential for maintaining basal keratinocyte identity and supporting epidermal differentiation.^[^ ^56^ ^]^ While CK14 is traditionally regarded as a basal marker, recent studies have shown its sustained expression during the prolonged remodeling phase in diabetic wounds, reflecting ongoing regenerative activity.^[^ ^57^ ^]^ Immunofluorescence staining revealed significantly elevated CK14 expression in the CA@Exos‐MNs + ES group, indicating enhanced re‐epithelialization and improved barrier restoration. Quantitative analysis further revealed a similar trend with the fluorescence images (Figure 7d).

The observed angiogenesis, neural regeneration, and re‐epithelialization at the wound site can be primarily ascribed to three key mechanisms. First, CA released from CA@Exos inhibits the formation of AGEs, thereby mitigating vascular and neural injuries in diabetic wounds. Second, the intrinsic angiogenic and neuroregenerative properties of CA@Exos contribute to the enhancement of neurovascular network reconstruction at the diabetic wound site. Third, ES enhances cellular uptake of CA@Exos and promotes cell migration, angiogenesis, and tissue remodeling, further accelerating the wound healing process. These results underscore the potential of the combined CA@Exos‐MNs and ES therapy as a promising strategy to counteract the detrimental effects of AGEs, while enhancing angiogenesis, neural regeneration, and re‐epithelialization, offering a novel approach for enhancing diabetic wound healing outcomes.

### 2.6 Transcriptomic Analysis of the CA@Exos‐MNs Combined with ES Promoting the Diabetic Wound Healing

**Figure 8:**
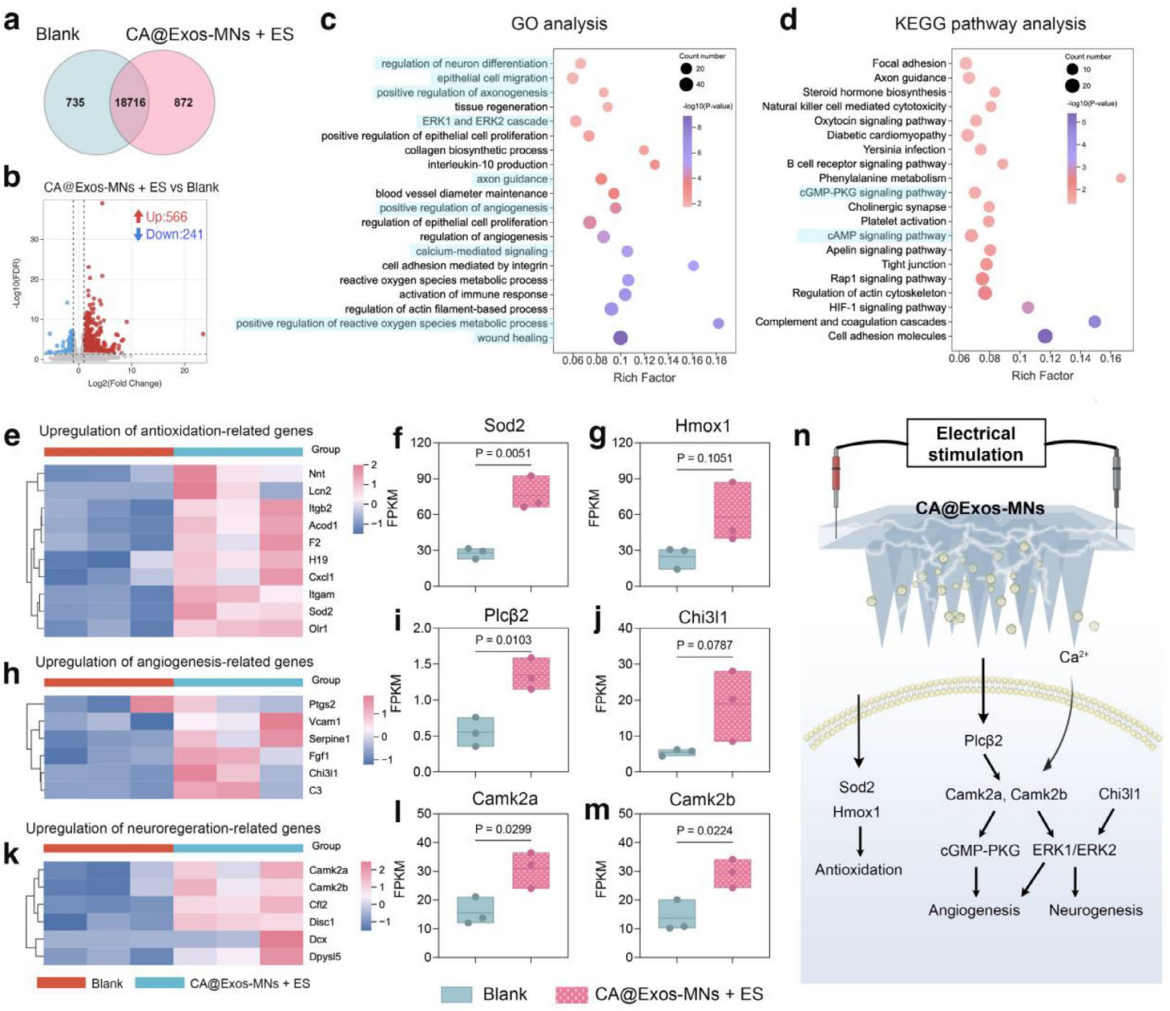
RNA‐sequence analysis of diabetic wound tissues. A) Venn diagram illustrating the number of genes in the comparison between Blank group and CA@Exos‐MNs + ES treated group. b) Volcano plot of the transcriptomic analysis of DEGs between the Blank group and CA@Exos‐MNs + ES treated group. c) GO classification of DEGs in CA@Exos‐MNs + ES group. d) KEGG enrichment pathway analysis of the identified DEGs. Heatmaps of significantly upregulated genes related to e) antioxidation, h) angiogenesis, and k) neurogenesis in the CA@Exos‐MNs + ES group as compared to the Blank group. Fragments per kilobase million (FPKM) of f) Sod2, g) Hmox1, i) Pclβ2, j) Chi3l1, l) Camk2a, and m) Camk2b (*n* = 3). n) Schematic illustration showing the potential mechanism underlying the enhancement of diabetic wound healing by the combination of CA@Exos‐MNs and ES. Data are presented as mean values ± SD. Comparisons were performed by unpaired two‐tailed Student's t test in (f, g, i, j, l, m). Differences were considered statistically significant at *p* < 0.05.

To further explore the mechanism underlying the enhancement of diabetic wound healing by the combination of CA@Exos‐MNs and ES, transcriptomic analysis was performed by employing RNA sequencing on skin wound tissues harvested at day 28. Venn diagram displayed that 18716 genes were found to be shared in the CA@Exos‐MNs + ES and Blank groups, with 872 genes uniquely expressed in CA@Exos‐MNs + ES group (**Figure** **8**a), suggesting the combination of CA@Exos‐MNs and ES induced a distinct gene expression profile compared to the Blank group. The volcano plot revealed 807 differentially expressed genes (DEGs) following CA@Exos‐MNs + ES treatment compared to the Blank group, of which 566 genes were upregulated, and 241 genes were downregulated (Figure 8b). Furthermore, GO enrichment analysis showed that the DEGs in CA@Exos‐MNs + ES group were enriched in biological processes such as wound healing, epithelial cell migration, positive regulation of ROS metabolic process, positive regulation of angiogenesis, positive regulation of calcium ion transport, ERK1 and ERK2 cascade, and axon guidance (Figure 8c). KEGG analysis revealed that, compared to the Blank group, DEGs in CA@Exos‐MNs + ES group were critically involved in axon guidance, cyclic adenosine monophosphate (cAMP) signaling pathway, Ras‐related protein 1 (Rap1) signaling pathway, and cGMP‐PKG signaling pathway (Figure 8d). These results highlight the potential of CA@Exos‐MNs + ES treatment in modulating key processes involved in mitigating oxidative stress and fostering neuroangiogenesis during the diabetic wound healing process.

Further, heatmap analysis of gene expression associated with antioxidation, angiogenesis, and neurogenesis between CA@Exos‐MNs + ES and Blank groups was performed. As shown in Figure 8e–g, antioxidation‐related genes (Sod2, Itgb2, Itgam, Acod1, Nnt, and H19) were markedly upregulated in CA@Exos‐MNs + ES group compared to the Blank group. Among them, Sod2 can effectively mitigate oxidative stress by accelerating the metabolic process of ROS,^[^ ^58^ ^]^ while Hmox1, a key enzyme in heme metabolism, reduces intracellular free radical levels and protects cells from oxidative stress.^[^ ^59^ ^]^ These outcomes indicate that the CA@Exos‐MNs + ES treatment not only enhances the expression of key crucial antioxidant genes, particularly Hmox1, but also modulates the metabolic process of ROS metabolic to alleviate the AGEs‐induced oxidative microenvironment. Additionally, CA@Exos‐MNs + ES treatment markedly upregulated the angiogenesis‐related genes (Vcam1, C3, Ptgs2, Chi3l1, and Fgf1) (Figure 8h–j) and the neurogenesis‐related genes (Cfl2, Camk2a, Camk2b, Disc1, Dcx, and Dpysl5) (Figure 8k–m). Among them, Chi3l1 activates the Erk1/2 signaling cascade, a critical pathway in promoting angiogenesis.^[^ ^60^ ^]^ Furthermore, activation of Plcβ has been reported to elevate intracellular Ca^2+^ levels, leading to the activation of calmodulin‐dependent protein kinase II (CaMKII), which triggered the Erk1/2 signaling cascade and the cGMP‐PKG signaling pathway, thereby fostering angiogenesis.^[^ ^60^, ^61^ ^]^ On the other hand, The CA@Exos‐MNs + ES treatment facilitated Ca^2+^ influx through positively modulating calcium ion transport, which activated the Ca^2+^ influx‐related gene (Camk2a and Camk2b), subsequently initiating the Erk1/2 cascade and effectively promoting neurogenesis. Notably, Camk2a and Camk2b are pivotal genes that synergistically drive angiogenesis and neurogenesis through activating Erk1/2 cascade.^[^ ^62^ ^]^ Above all, these findings suggest that CA@Exos‐MNs + ES treatment effectively alleviates oxidative stress by elevating key antioxidant gene expressions and expediting the metabolic breakdown of ROS, while promoting neuroangiogenesis via Erk1/2 cascade and cGMP‐PKG signaling pathway, thereby expediting the healing process of the diabetic wounds (Figure 8n).

## 3 Conclusion

In this study, we developed a multifunctional strategy termed CA@Exos‐MNs combined with electrochemical stimulation to reshape neurovascular niches in diabetic wounds under hyperglycemic microenvironments. By engineering *Saccharina japonica*‐derived exosomes for efficient CA loading, we validated the multiple bioactivities of CA@Exos in counteracting glycation, oxidative stress, and inflammation, while enhancing crosstalk between Schwann cells and HUVECs. CA@Exos were loaded onto the needle tip of conductive microneedles (ASA/pCNTs‐based MNs), which enabled deep tissue delivery and sustained release of CA@Exos, with intrinsic electrical conductivity significantly augmenting cellular uptake of CA@Exos. In diabetic rat wound models, this combinatorial approach reprogrammed macrophage polarization and reshaped the inflammatory milieu, concurrently suppressing AGE formation and restoring neurovascular networks. Transcriptomic profiling revealed that the therapy activated Camk2a/Camk2b‐mediated Erk1/2 and cGMP‐PKG signaling cascades, alleviating oxidative stress and promoting neural‐vascular regeneration. Importantly, the synergistic effects of electrical stimulation and CA@Exos‐MNs not only promoted re‐epithelialization but also established a self‐amplifying loop for oxidative homeostasis and neuroangiogenesis. This work proposes a paradigm‐shifting therapeutic platform for diabetic wound healing via spatiotemporal immunometabolic modulation and multi‐signaling pathways coactivation, offering a blueprint for precision intervention in chronic inflammatory disorders. In summary, this study established a bio‐electroceutical interface by synergizing engineered exosome‐derived biological signals with electroconductive microneedle‐delivered electrical cues, achieving dual‐pathway reprogramming of the diabetic wound microenvironment and accelerating wound healing.

## 4 Experimental Section

The preparation and characterization methods are provided in the Supporting Information.

### Animal Ethics Statement

All animal experiments were approved by Ocean University of China Animal Laboratory Animal Ethics Committee (approval numbers: OUC‐SMP‐2024‐03‐08 and OUC‐SMP‐2025‐02‐12).

## Conflict of Interest

The authors declare no conflict of interest.

## Supporting information



Supporting Information

## Data Availability

The data that support the findings of this study are available from the corresponding author upon reasonable request.
