# Supplementary material for: Conductive Microneedles Loaded With Polyphenol‐Engineered Exosomes Reshape Diabetic Neurovascular Niches for Chronic Wound Healing
Source: Adv Sci (Weinh). 2025 Aug 26;12(43):e07974. doi: 10.1002/advs.202507974 (PMC12631848; doi:10.1002/advs.202507974)
Supplement: Supplementary file 1 — Supporting Information [file ADVS-12-e07974-s001.docx]

Supporting Information

**Conductive Microneedles Loaded with Polyphenol-Engineered Exosomes Reshape Diabetic Neurovascular Niches for Chronic Wound Healing**

Di Liu, Jingxian Gao, Xueling Wu, Xinxin Hao, Wenxiu Hu, Lu Han*

D. Liu, J. Gao, X. Wu, X. Hao, W. Hu, Pro. L. Han

Key Laboratory of Marine Drugs

Ministry of Education

School of Medicine and Pharmacy

Ocean University of China

Qingdao 266003, China.
E-mail: hanlu@ouc.edu.cn

Pro. L. Han

Laboratory for Marine Drugs and Bioproducts

Qingdao Marine Science and Technology Center

Qingdao 266237, China.

**Materials**

Bicinchoninic acid (BCA) kit, 3,3′-dioctadecyloxacarbocyanine perchlorate (DiO), 1,1'-dioctadecyl-3,3,3',3'-tetramethylindodicarbocyanine,4-chlorobenzenesulfonate salt (DiD), S100 beta rabbit monoclonal antibody (S100β), and FITC-conjugated goat anti-rabbit IgG were purchased from Beyotime Biotechnology Co., Ltd (China). Agarose and single-walled carbon nanotubes (SWCNTs) were purchased from Adamas Co., Ltd. (China). Octenyl succinic anhydride (OSA), 4-dimethylaminopyridine (DMAP), caffeic acid (CA), and 2,2-diphenyl-1-picrylhydrazyl (DPPH) were purchased from Macklin Biochemical Technology Co., Ltd (China). Dopamine hydrochloride and dimethyl sulfoxide (DMSO) was purchased from Aladdin Biochemical Technology Co., Ltd (China). Hydrogen peroxide solution (30%) was purchased from Xilong Science Co., Ltd. (China). Human umbilical vein endothelial cells (HUVECs) were bought from Chinese Academy of Sciences Cell Bank, Shanghai, China. Schwann cells were bought from Pricella Biotechnology Co., Ltd (China). Mouse fibroblast cells (L929 cells) and mouse leukemia cells of monocyte macrophage (RAW264.7) cells were obtained from the cell bank of the Type Culture Collection Committee of the Chinese Academy of Sciences. Dulbecco's modified Eagle's medium (DMEM), Trypsin-EDTA (0.25%), RNA rapid extraction solution, water nuclease-free, SweScript All-in-One RT SuperMix for qPCR (One-Step gDNA Remover), 2×Universal Blue SYBR Green qPCR Master Mix, and phosphate buffered saline (PBS) were purchased from Servicebio Biotechnology Co., Ltd (China). Fetal bovine serum (FBS) was purchased from Wisent corporation (Canada). Cell counting kit-8 (CCK-8) was purchased from SparkJade (Shandong, China). Lipopolysaccharides (LPS), bovine serum albumin (BSA), 4,6-diamino-2-phenyl indole (DAPI), 2',7'-dichlorodihydrofluorescein diacetate (DCFH-DA), penicillin-streptomycin-gentamicin, 4% paraformaldehyde, Triton X-100, and live/dead staining kits were purchased from Solarbio Science & Technology Co., Ltd (China). TraKine^TM^ F-actin Staining Kit (Orange Fluorescence) was obtained from Abbkine (Wuhan, China).

**Experimental Section**

**Isolation, purification, and characterization of** ***saccharina japonica*****-derived exosomes (Exos)**

*Saccharina japonica*-derived exosomes were isolated and purified by continuous differential centrifugal and sucrose density gradient centrifugation. Firstly, *saccharina japonica* obtained from a seafood market in Qingdao was washed with deionized (DI) water and ground into a blender to obtain juice. Secondly, the juice was ﬁltered into a new container through two layers of gauze. Thirdly, the filtrate was subjected to three successive centrifugations at 4 °C: 1,000 g for 10 min, 3,000 g for 30 min, and 10,000 g for 30 min. The supernatant was collected and filtered through 0.22 μm syringe-driven filter units. Fourthly, the filtrate was ultracentrifuged at 100,000 g for 70 min at 4 °C using an ultracentrifuge (L100XP, Beckman, USA). For purification of exosomes, the resulting precipitate was resuspended in phosphate-buffered saline (PBS) and transferred to a discontinuous sucrose gradient (15%, 30%, 45%, and 60% (w/v)), followed by ultracentrifugation at 100,000 g for 70 min at 4 °C. The bands between 30/45% layers were collected and further ultracentrifuged at 100,000 g for 70 min at 4 °C. Finally, the resulting pellets of Exos were resuspended in PBS and stored at -80 °C until use.

Transmission electron microscopy (TEM, JEM-1200EX, JEOL, Japan) was used to characterize the morphology of Exos. The exosomal protein concentration was assessed using a bicinchoninic acid (BCA) protein quantification kit. The concentration of Exos (particles mL^-1^) was measured uding a Nanocoulter S (RESUN-S01, Resuntech, China).

**Exos RNA library construction and sequencing**

Exosomes extracted from three distinct batches of *Saccharina japonica* were used in this study. Total RNA was extracted using Trizol reagent (Invitrogen, Carlsbad, CA, USA) according to manufacturer’s protocol and quantified using a NanoDrop ND-1000 (NanoDrop, Wilmington, DE, USA). Approximately 5 µg of total RNA was used to deplete ribosomal RNA according to the manuscript of the Ribo-Zero™ rRNA Removal Kit (Illumina, San Diego, USA). The remaining RNA was fragmented and reverse-transcribed into cDNA, followed by U-labeled second-strand synthesis. An A-base was added to the blunt ends for ligation with indexed adapters containing a T-base overhang. Single-or dual-index adapters were ligated to the fragments, and size selection was performed with AMPureXP beads. After the heat-labile UDG enzyme treatment of the U-labeled second-stranded DNAs, the ligated products were amplified with PCR. The average insert size for the final cDNA library was 300 bp (± 50 bp). Paired-end sequencing was conducted on an Illumina HiSeq 4000 (LC Bio, China) following standard protocols.

**Target gene prediction of miRNAs contained in Exos and functional analysis**

Identification of known miRNAs was performed with miRBase 22.1. To predict the genes targeted by most abundant miRNAs, two computational target prediction algorithms (TargetScan (5.0) and Miranda (3.3a), TargetScan_score ≥ 50 and miranda_Energy < 10) were used to identify miRNA binding sites. Finally, the data predicted by both algorithms were combined and the overlaps were calculated. GO/KEGG enrichment analysis provides all GO terms/KEGG pathways that significantly enriched in miRNA target genes comparing to the genome background. All miRNA target genes were mapped to GO terms/KEGG pathways in the Gene Ontology database (http://www.geneontology.org/)/Kyoto Encyclopedia of Genes and Genomes (https://www.kegg.jp/), gene numbers were calculated for every term/pathway, significantly enriched GO terms/KEGG pathways in miRNA target genes comparing to the genome background were defined by hypergeometric test.

**In vitro cytocompatibility of Exos**

The cytocompatibility of Exos was evaluated by using HUVECs and Schwann cells, which were cultured in complete DMEM medium supplemented with 10% FBS and 1% penicillin-streptomycin-gentamicin solution at 37 °C in 5% CO_2_. The Exos were sterilized by filtration through a 0.22 μm syringe-driven filter unit. Firstly, cells were seeded in 96-well cell-culture plates at a density of 5 × 10^3^ cells per well. After 12 h, the complete DMEM medium was replaced with Exos-supplemented complete DMEM medium at varying concentrations (0, 10, 25, 50, and 75 µg mL^-1^). For each group, there were 4 parallel samples. After 24 h of co-culture, the medium was removed, and 100 μL of serum-free DMEM along with 10 μL of CCK-8 solution was added to each well. After incubation at 37 °C for 2 h, absorbance of each well at 450 nm was measured using a microplate reader (Spark, TECAN, Switzerland).

**Cellular uptake of Exos**

Prior to cell culturing, Exos were labeled with DiO following the manufacturer’s instructions. 5 μL of DiO probe was added into 0.15 mL of Exos solution (1.4 mg mL^-1^). After incubation at room temperature in dark for 30 min, the above mixture was ultracentrifuged at 100,000 g for 70 min at 4 °C and washed for 3 times with PBS to remove residual dye. HUVECs or Schwann cells were seeded at a density of 8 × 10^5^ cells in glass bottom cell culture dishes. After 12 h of culturing, the complete DMEM medium was replaced with DiO-labeled-Exos-supplemented complete DMEM medium (50 µg mL^-1^), and cells were co-cultured for 6 h. For visualization, F-actin was stained with phalloidin, and the nuclei were stained with 4,6-diamino-2-phenyl indole (DAPI). The stained cells were subsequently observed using a confocal laser scanning microscope (CLSM, TCS SP8 STED 3X, Leica, Germany).

**Tube formation assay**

Matrigel solution was thawed overnight at 4 °C and then added (80 µL per well) into 24-well cell-culture plates, followed by incubation at 37 °C for 1 h to allow gel solidification. HUVECs (1.5 × 10^5^ cells per well) were seeded into the Matrigel-precoated wells in a suspension containing Exos (50 µg mL^-1^). After 6 h of co-culture, HUVECs were stained with Calcein-AM, and tube formation was observed using CLSM (TCS SP8 STED 3X).

**Real-time quantitative PCR (RT-qPCR) analysis and enzyme-linked immunosorbent assay (ELISA) assay**

HUVECs were initially seeded at a density of 1 × 10^5^ cells per well in 6-well cell culture plates. After 12 h of culturing, the complete DMEM medium was replaced with Exos-supplemented complete DMEM medium (50 µg mL^-1^). After co-culture for 6 days, the HUVECs were collected. For each group, there were 3 parallel samples. For RT-qPCR analysis, total RNA of cells was extracted using chloroform substitute and quantiﬁed by measuring the absorbance at 260 nm and 280 nm using a spectrophotometer (NanoDrop2000, Thermo Fisher Scientiﬁc, USA). RNA was reverse transcribed using SweScript All-in-One RT SuperMix for qPCR (One-Step gDNA Remover) to generate cDNA. Real-time PCR was performed with 2×Universal Blue SYBR Green qPCR Master Mix on a Real-Time PCR Detection System (CFX Connect, Bio-rad, USA). Gene expression levels were quantified using the comparative Ct method (ΔΔCt), normalizing target gene expression to a reference gene. Detailed primer sequences were listed in Table S1.

Schwann cells were initially seeded at a density of 1 × 10^5^ cells per well in 6-well cell-culture plates. After 12 h, the complete DMEM medium was replaced with Exos-supplemented complete DMEM medium (50 µg mL⁻¹). After co-cultured for 6 days, the Schwann cells were collected. For each group, there were 3 parallel samples. Detailed primer sequences used in RT-qPCR analysis were listed in Table S2.

For ELISA assay, the culture medium of Schwann cells co-cultured with Exos (50 µg mL^-1^) for 6 days was collected, and the expression levels of brain-derived neurotrophic factor (BDNF) were tested according to the manufacturer’s instructions, followed by quantification using a microplate reader (FRT6100, Rayto Life and Analytical Sciences Co., Ltd, China) at 450 nm.

**Immunoﬂuorescence staining of S100 beta (S100β) in Schwann cells**

Firstly, Schwann cells were seeded at a density of 4 × 10^5^ cells per well in glass bottom cell culture dishes. After 12 h of culturing, the complete DMEM medium was replaced with Exos-supplemented complete DMEM medium (50 µg mL^-1^), and the cells were co-cultured for 3 days. Secondly, the Schwann cells were fixed with 4% paraformaldehyde for 15 min at 4 °C, incubated with 1 mL of Triton X-100 (0.2%) for 20 min at room temperature, and then incubated with 1 mL of BSA solution (3%) for 1 h. Thirdly, the Schwann cells were incubated with S100 beta rabbit monoclonal antibody (S100β, 1:250) overnight at 4 °C, followed by incubation with FITC-conjugated goat anti-rabbit IgG (1:200) for 2 h at room temperature. Finally, nuclei were stained with DAPI for 15 min at 37 °C, and stained cells were visualized using a CLSM (TCS SP8 STED 3X).

**Loading** **caffeic acid (CA) into Exos (CA@Exos)**

CA was loaded into the exosomes by a co-incubation method. Briefly, CA was dissolved in PBS (pH7.4) at a concentration of 1 mg mL^-1^. Exos were dispersed in PBS (pH 7.4) at a concentration of 0.6 mg mL^-1^. Then, CA solution and Exos solution were mixed at a volume ratio of 1:1 and incubated for 2 h at 37 °C in a shaker (HNY-200F, Honour, China). To remove free or surface-adsorbed CA, the mixture was ultracentrifuged at 10,000 g for 70 min, and the resulting precipitation was washed with PBS for 3 times to obtain CA@Exos.

**Characterization of** **CA@Exos**

The Zeta potential values of Exos and CA@Exos were measured by using a dynamic light scattering (DLS, ZEN3700, Malvern, England). The marker proteins (CD9 and CD63) of Exos and CA@Exos were detected by a Flow NanoAnalyzer (NanoFCM INC, Resuntech, China) using anti-CD9 and anti-CD63 antibodies, according to manufacturer’s protocols.

After co-incubation of CA and Exos solutions for 2 h, the absorbance at 312 nm of CA in the supernatant after centrifugation of the mixture was measured by using a UV-Vis spectrophotometer (TU-1810, PERSEE, China). A standard calibration curve was established by measuring the absorbance of CA at various known concentrations. Three parallel samples were used in the test. The encapsulating efficiency (EE) and loading efficiency (LE) were calculated using the equations as follows:

$$\begin{aligned} EE=\frac{w_{1}}{w_{2}}\times100\%\#(Equation S1) \end{aligned}$$

$$\begin{aligned} LE=\frac{W_{1}}{W_{3}}\times100\%\#(Equation S2) \end{aligned}$$

Where W_1_ represents the weight of loaded CA, W_2_ represents the feeding weight of CA, and W_3_ represents the weight of CA@Exos.

The chemical structures of CA, Exos, and CA@Exos were analyzed using Fourier-transform infrared (FTIR) spectroscopy (Nicolet iS50 FTIR, Thermo Scientific, USA) in the range of 4000-400 cm^-1^.

**Cumulative** **release curves of CA@Exos in vitro**

1 mL of CA@Exos solution (1 mg mL^-1^) was placed in dialysis bags with a molecular weight cutoff (MWCO) of 3500 Da. Then the dialysis bags were immersed in 30 mL of PBS containing 0.5% Tween 80 or PBS containing 0.5% Tween 80 and 0.1% Triton X-100 and incubated in a shaker (HNY-200F) at 170 rpm and 37 °C. At predetermined time intervals (0.5, 1, 2, 4, 8, 12, 24, 48, and 72 h), 3 mL of the release medium was withdrawn and replenished with an equal volume of fresh PBS containing 0.5% Tween 80 or PBS containing 0.5% Tween 80 and 0.1% Triton X-100. The absorbance of withdrawn solution at 312 nm was detected using a UV-Vis spectrophotometer (TU-1810). Three parallel samples were used in the test. The cumulative release of CA was calculated using equation S3:

$$\begin{aligned} The cumulative release \left( \% \right)=\left( V_{e}\sum_{1}^{n-1} C_{i}+V_{o}C_{n} \right)/m\times100\#(Equation S3) \end{aligned}$$

Where V_e_ represents the volume of withdrawn solution; C_i_ represents the concentration of CA at each timepoint; V_0_ represents the initial volume of PBS containing 0.5% Tween 80 or PBS containing 0.5% Tween 80 and 0.1% Triton X-100; C_n_ represents the concentration of CA at the first time point (0.5 h); m represents the total amount of CA loaded in CA@Exos.

**In vitro cytocompatibility of CA@Exos**

The cytocompatibility of CA@Exos was evaluated by using Schwann cells, L929 cells, and HUVECs. L929 cells were cultured in DMEM medium supplemented with 10% FBS and 1% penicillin-streptomycin-gentamicin solution at 37 °C in 5% CO_2_. CA@Exos were sterilized by filtration through a 0.22 μm syringe-driven filter unit. The cells were seeded at a density of 5 × 10^3^ cells per well in 96-well cell-culture plates. After 12 h of culturing, the complete DMEM medium was replaced with Exos-supplemented complete DMEM medium at varying concentrations (10, 25, 50, 75 µg mL^-1^). For each group, there were 4 parallel samples. After 24 h of co-culture, the medium was removed from cells, and 100 μL of serum-free DMEM along with 10 μL of CCK-8 solution was added to each well. After incubation at 37 °C for 2 h, absorbance of each well at 450 nm was measured using a microplate reader (Spark).

**Preparation of advanced glycation end products-bovine serum albumin (AGE-BSA)**

BSA (50 mg mL^-1^ in PBS) and D-glucose (90 mg mL^-1^ in PBS) were co-incubated at 37 °C for 3 months to induce glycation. The resulting mixture was dialyzed with a dialysis bag (molecular weight cutoff: 1000 Da) for 12 h at 4 °C to obtain AGE-BSA. The concentration of AGE-BSA was determined by using a BCA protein quantification kit. The molecular weight change of AGE-BSA was determined by sodium dodecyl sulphate-polyacrylamide gel electrophoresis (SDS-PAGE) using a gel imaging system (W1000-plus, Servicebio, China).

**Antiglycative activity of CA@Exos**

To characterize the antiglycative activity of CA@Exos, BSA (50 mg mL^-1^ in PBS) and D-glucose (90 mg mL^-1^ in PBS) were firstly mixed. Then, 1 mL of mixed solution was co-incubated with PBS, Exos or CA@Exos (50 µg mL^-1^) at 37 °C in the dark. After predetermined time points (6, 9, and 14 days), the mixture was ultracentrifuged, and the fluorescence intensity of supernatant was measured using a fluorescence spectrometer (λ_ex_ = 370 nm / λ_em_ = 440 nm) (FLS1000, Edinburgh Instruments Ltd., UK).

To evaluate the interaction between CA and AGE-BSA, 1 mL of AGE-BSA solution (200 µg mL^-1^) was incubated with PBS, Exos or CA@Exos (50 µg mL^-1^) at 37 °C in dark. After incubation for 3 days, the absorbance of samples was detected using a UV-Vis spectrophotometer (TU-1810).

**Molecular docking analysis**

Molecular docking between AGEs and CA was performed using Schrödinger software. AGEs structures were modeled by site-specific mutation of bovine serum albumin (BSA, PDB ID: 4 F5S),^[1]^ in which selected arginine residues (Arg194, Arg196, Arg198, and Arg217) were mutated to argpyrimidine, a methylglyoxal-derived AGE formed via cyclocondensation with L-arginine. Additionally, the lysine residue (Lys204) of BSA was mutated to carboxyethyllysine (CEL), a representative lysine-derived AGE formed via non-enzymatic glycation. The modified structures were constructed and optimized using Gaussian, followed by Ligand Preparation to refine the geometry and assign appropriate partial charges for docking. Binding affinity energies between CA and AGEs were calculated based on the equation ∆G=$-RTlnk_{d}$, indicating strong interaction.

**Protective effect of CA@Exos on Schwann cells against oxidative stress**

To evaluate the protective effect of CA@Exos against AGE-BSA or H_2_O_2_-induced oxidative stress, Schwann cells (6 × 10^5^ cells per dish) were seeded in glass bottom cell culture dishes, and 1 mL of complete DMEM medium was added into each dish. After 12 h of culturing, the complete DMEM medium was replaced with CA@Exos-supplemented complete DMEM medium (50 µg mL^-1^) in the presence of AGE-BSA (200 µg mL^-1^) or H_2_O_2_ (50 μM). After o-culture for 6 h, the medium was replaced with complete DMEM medium containing 2′,7′-dichlorodihydrofluorescein diacetate (DCFH-DA) and incubated for 30 min in dark, followed by three times washes with PBS. Untreated cells were set as negative control, while cells treated with AGE-BSA or H_2_O_2_ were set as positive control. Finally, intracellular fluorescence was observed using CLSM (TCS SP8 STED 3X).

For the flow cytometry analysis of CA@Exos against H_2_O_2_-induced oxidative stress, stained cells were collected, washed, and resuspended in 300 μL of PBS, followed by assessment using flow cytometry (Gallios, Beckman Coulter, USA).

**Evaluation of Exos recycling**

Firstly, Schwann cells were seeded at a density of 3 × 10^4^ cells per well in 48-well cell-culture plates and cultured for 12 h in complete DMEM medium. Secondly, the complete DMEM medium was replaced with complete DMEM medium containing DiO-labeled-Exos (50 µg mL^-1^) for 24 h to allow internalization. Thirdly, the culture medium was replaced with complete DMEM medium containing CA@Exos (50 µg mL^-1^) in the presence of AGE-BSA (200 µg mL^-1^). The untreated cells were set as negative control, while cells treated with AGE-BSA were set as positive control. After 12 h of co-culture, DiO fluorescence signals were detected using a fluorescence spectrometer (FLS1000). Three samples were used for the test.

**Anti-inflammatory activity of CA@Exos**

RAW264.7 cells were seeded at a concentration of 8 × 10^5^ cells per well in glass bottom cell culture dishes and cultured for 12 h in complete DMEM medium. Afterward, the complete DMEM medium was replaced with complete DMEM medium containing LPS (4 µg mL^-1^) to induce inflammatory responses for 24 h. Then, the culture medium was removed and complete DMEM medium containing CA@Exos (50 µg mL^-1^) were added to the dishes. After 24 h of co-culture, the RAW264.7 cells were firstly fixed with 4 % paraformaldehyde for 20 min, permeabilized with 0.1 % Triton X-100 for 30 min, then washed two times with PBS. Secondly, the cells were blocked with BSA solution (50 mg mL^-1^) followed by incubated with iNOS rabbit polyclonal antibody (31265ES60, Yeasen, 1:100) and anti-mouse CD206 (141701, Biolegend, 1:100) overnight at 4 °C. Thirdly, the cells were washed with PBS for 3 times and incubated with FITC-labeled goat anti-rabbit lgG (H+L) (A0562, Beyotime, 1:100) and AlexFluor 594 Donkey Anti-Rat lgG (H+L) (34412ES60, Yeasen, 1:100) for 4 h. Nuclei were stained with DAPI for 20 min at 37 °C. Finally, the stained cells were observed by CLSM (TCS SP8 STED 3X).

**Scratch assays**

L929 cells were used to evaluate the effect of CA@Exos on cell migration. Firstly, L929 cells were seeded in 6-well cell-culture plates and cultured until reaching 90% confluence. Secondly, a scratch line was created with pipette tip on the cell monolayer and the cells were washed withby PBS for three times to remove the unattached cells. Thirdly, the FBS-free DMEM medium with CA@Exos (50 µg mL^-1^) was added into the 6-well cell-culture plate. After co-culture for 24 h and 48 h, cell migration was observed using an optical microscope (CKX53, OLYMPUS, Japan).

**Indirect co-culture system for Schwann cells and HUVECs**

To prepare Schwann cells-conditioned medium (Schwann cells^CM^), Schwann cells were seeded at a density of 3 × 10^5^ cells per well in 6-well cell-culture plates for 12 h, followed by replacing complete DMEM medium with complete DMEM medium containing CA@Exos (50 µg mL^-1^). After co-culture for 24 h, the culture medium was collected and centrifuged (1200 rpm, 5 min) to obtain supernatants. The resulting supernatant was mixed with fresh complete DMEM medium at a 1:1 volume ratio and used as conditioned medium (Schwann cells^CM^). To quantify the residual CA@Exos in Schwann cells^CM^, Schwann cells were seeded at a density of 3 × 10^5^ cells per well in 6-well cell-culture plates for 12 h, followed by replacing complete DMEM medium with complete DMEM medium containing DiO-labeled-CA@Exos (50 µg mL^-1^). After co-culture for 24 h, the culture medium was collected and centrifuged (1200 rpm, 5 min) to obtain supernatants. The fluorescence intensity of supernatants was measured by using a microplate reader (Spark). Three parallel samples were used for the test.

To prepare HUVECs-conditioned medium (HUVECs^CM^), HUVECs were seeded at a density of 3 × 10^5^ cells per well in 6-well cell-culture plates for 12 h, followed by replacing complete DMEM medium with complete DMEM medium containing CA@Exos (50 µg mL^-1^). After co-culture for 24 h, the culture medium was collected and centrifuged (1200 rpm, 5 min) to obtain supernatants. The resulting supernatant was mixed with fresh complete DMEM medium at a 1:1 volume ratio and used as conditioned medium (HUVECs^CM^).

To assess the effect of Schwann cells^CM^ on tube formation in HUVECs, 80 µL of Matrigel solution was added into each well of 24-well cell-culture plates and incubated at 37 °C for 1 h to allow gel solidification. HUVECs (1.5 × 10^5^ cells per well) were seeded into the Matrigel-precoated wells in the presence of Schwann cells^CM^. After 6 h of co-culture, HUVECs were stained with Calcein-AM and tube formation was observed using CLSM (TCS SP8 STED 3X).

For RT-qPCR analysis of angiogenesis-related gene expression (CD31 and VEGF), HUVECs were initially seeded at a density of 5 × 10^5^ cells per well in 6-well cell-culture plates. After 12 h of co-culture, the complete DMEM medium was replaced with Schwann cells^CM^. After co-culture for 3 days, HUVECs were collected, and the expression of genes was analyzed by RT-qPCR. For each group, there were 3 parallel samples. Detailed primer sequences are listed in Table S3.

For the RT-qPCR analysis of neurogenic-related gene expression (BDNF, S100β, and NGF), Schwann cells were initially seeded at a density of 5 × 10^5^ cells per well in 6-well cell-culture plates. After 12 h of co-culture, the complete DMEM medium was replaced with HUVECs^CM^. After co-culture for 3 days, Schwann cells were collected, and the expression of genes was analyzed by RT-qPCR. For each group, there were 3 parallel samples. Detailed primer sequences are listed in Table S2.

**Synthesis of** **octenyl succinic anhydride-grafted agarose (ASA)**

Firstly, 2 g of agarose was dissolved in 30 mL of DMSO under stirring at 90 °C and then the agarose solution was cooled down to 40 °C. Secondly, 0.2 g of 4-dimethylaminopyridine (DMAP) and 1.5 g of octenyl succinic anhydride (OSA) were added to the agarose solution in turn and the reaction continued for 3.5 h at 40 °C. Thirdly, the reaction solution was slowly added into deionized (DI) water under continuous stirring. Finally, the ASA was obtained by lyophilization.

**Synthesis of polydopamine-decorated-single-walled carbon nanotubes (pCNTs)**

Firstly, 0.2 g of single-walled carbon nanotubes (SWCNTs) was dispersed in 100 mL of DI water and ultrasonicated for 2 h. Secondly, 0.1 g of dopamine hydrochloride was added into the above SWCNTs dispersions and then the pH of the solution was adjusted to 11 using NaOH solution. Finally, the solution was stirred at room temperature (25 °C) for 24 h. pCNTs were finally obtained by centrifugation at 10,000 rpm for10 min, followed by repeated washing with DI water and lyophilization.

**Structure characterizations of agarose, ASA,** **SWCNTs, and pCNTs**

The hydrogen spectrum nuclear magnetic resonance (^1^H NMR) spectrum of ASA was characterized by using d6-DMSO as solvent on a DD2 600 MHz spectrometer (JNM-ECZ600R/S1, JEOL, Japan). The chemical structures and functional groups of agarose, ASA, SWCNTs, and pCNTs were analyzed by FTIR (Nicolet iS50) in the range of 4000-400 cm^-1^.

**Fabrication of the pCNTs-ASA microneedles**

Typically, 80 mg of ASA was dissolved in 1 mL of DMSO at 60 °C, and then 4 mg of pCNTs were dispersed into ASA solution. Next, 400 µL of above mixture was added in a PDMS mold (14 mm × 14 mm × 1.5 mm, with a needle height of 600 μm and a 320 μm diameter at the base). The mixture in the mold was centrifugated at 4,000 rpm for 10 min to ensure that the needle tips were filled. After that, the mold with mixture was dried in an oven at 60 °C for 8 h to obtain the pCNTs-ASA microneedles (pCNTs-ASA MNs). For comparison, pCNTs-ASA MNs with varying contents of pCNTs were fabricated as listed in Table S4.

**Mechanical properties of** **pCNTs-ASA MNs**

The compressive forces of the pCNTs-ASA MNs with varying contents of pCNTs were tested by using a universal mechanical testing machine (CMT5105, SUST, China). The pCNTs-ASA MNs were placed on the fixed platform and a loading rate of 0.2 mm min^-1^ was applied until a maximum strain of 80% was reached, during which the compressive curves was recorded.

**Electroconductive characterization of** **pCNTs-ASA films**

To facilitate the test of conductivity of pCNTs-ASA MNs with varying contents, pCNTs-ASA solution was poured onto the surface of the glass dish and dried in an oven at 60 °C for 8 h to obtain the pCNTs-ASA films. The conductivity of pCNTs-ASA films was measured using a standard four-point probe system (ST2263, SuzhouJinggeElectronicCo., LTD) with 10 mm in length, 10 mm in width, and 0.6 mm in thickness. Cyclic voltammetry was performed on pCNTs-ASA film or Au foil (exposed area of 1 cm^2^) in PBS using platinum (Pt) foil as a counter electrode and silver/silver chloride (Ag/AgCl) as a reference electrode. The measurements were conducted on an electrochemical workstation (CHI660E, CH Instruments, USA) at a scan rate of 0.15 mV s^-1^. Square-wave voltage pulses were applied on pCNTs-ASA films from -0.5 to 0.5 V with each duration of 50 ms and the corresponding current was simultaneously recorded.

**Anti-oxidative property of pCNTs-ASA MNs**

To characterize the DPPH radical scavenging ability, 2 mg of pCNTs-ASA MNs with varying contents of pCNTs was immersed in 1 mL of DPPH solution (0.04 mg mL^-1^ in ethanol) at 37 °C in dark. Pure DPPH solution was used as a control group. After a predetermined time, the supernatant absorbance at 516 nm was tested by microplate reader (Spark). Three parallel samples were used for the test. The DPPH radical scavenging efficiency is calculated according to equation S4.

$$\begin{aligned} DPPH radical scavenging \left( \% \right)=\frac{A_{B}-A_{S}}{A_{B}}\times100\#(Equation S4) \end{aligned}$$

Where A_B_ represents the absorbance of the pure DPPH solution and A_S_ represents the absorbance of the DPPH solution after incubated with pCNTs-ASA MNs with varying contents of pCNTs.

**Preparation and characterization of** **CA@Exos loaded pCNTs-ASA MNs (****CA@Exos-MNs)**

CA@Exos-MNs was prepared by physical adsorption method. Briefly, MNs were immersed in 400 µL of CA@Exos dispersion (500 µg mL^-1^ in PBS) for 12 h at 4 °C. PBS was used to wash off the free CA@Exos from the surface of MNs. The encapsulation efficiency of CA@Exos on CA@Exos-MNs was evaluated according to equation S5:

$$\begin{aligned} The encapsulation efficiency \left( \% \right)=\frac{m_{i}-m_{s}}{m_{i}}\times100\#(Equation S5) \end{aligned}$$

Where m_i_ represents the initial amount of CA@Exos; m_s_ represents the amount of CA@Exos in supernatant after co-incubation with MNs.

For scanning electron microscopy (SEM, JSM-840, JEOL, Japan) examination, MNs and CA@Exos-MNs were lyophilized using a lyophilizer. Prior to SEM imaging, the lyophilized MNs and CA@Exos-MNs were coated with a thin layer of gold via sputter coating. The distribution of DiO-labeled-CA@Exos on the MNs was observed by a CLSM (TCS SP8 STED 3X). The mechanical properties and conductivity of CA@Exos-MNs were characterized following the methods described above.

**In vitro** **cytocompatibility of** **MNs and CA@Exos-MNs**

The cytocompatibility of MNs and CA@Exos-MNs was assessed using Schwann cells. To prepare the extract solution (4 mg mL^-1^), MNs or CA@Exos-MNs were immersed in 1 mL of complete DMEM medium for 24 h. For live/dead staining assay, Schwann cells were seeded at a density of 1.5 × 10^5^ cells per well in glass bottom cell culture dishes. After the Schwann cells were attached on the glass bottom cell culture dishes, the complete DMEM medium was replaced with 1 mL of extract solution. After co-culture for 3 days, the culture medium was removed, and Schwann cells were incubated with calcein AM (1 μL mL^-1^) and propidium iodide (3 μL mL^-1^) for 30 min, then observed using a CLSM (TCS SP8 STED 3X).

The cell viability was also quantified by a CCK-8 assay. Schwann cells were seeded at a density of 5 × 10^3^ cells per well in 48-well cell-culture plates. After 12 h of culture, the complete DMEM medium was replaced with extract solution. After 1, 3, and 5 days of co-culture, the medium was removed from cells, and 300 μL of serum-free DMEM along with 30 μL of CCK-8 solution was added to each well. After incubation at 37 °C for 2 h, absorbance of each well at 450 nm was measured using a microplate reader (Spark). For each group, there were 5 parallel samples.

**Electrical stimulation (ES) facilitates the cellular uptake of CA@Exos**

Schwann cells were seeded at a density of 5 × 10^4^ cells per well in 48-well cell-culture plates coated with cell culture slides. After Schwann cells were attached on the cell culture slides, DiO-labeled-CA@Exos-MNs were gently covered onto the cells and incubated for 1.5 h. Subsequently, an ES potential (400 mV) was applied for 0.5 h using a home-made high throughput device. The cells without treatment were set as control group. The cells were then stained with phalloidin and DAPI for CLSM (TCS SP8 STED 3X) imaging. Additionally, the amount of CA@Exos in culture medium was detected using a BCA kit. For each group, there were 3 parallel samples. The uptake ratio of CA@Exos was calculated as following equation S6:

$$\begin{aligned} Uptake ratio \left( \% \right)=\frac{m_{1}-(m_{2}-m_{c})}{m_{1}}\times100\#(Equation S6) \end{aligned}$$

Where m_1_ represents the amount of CA@Exos on MNs; m_2_ represents the amount of CA@Exos in culture medium in group with ES or without ES; m_c_ represents the amount of CA@Exos in culture medium in control group.

In addition, the effect of 400 mV direct current used on cell viability was evaluated. Briefly, Schwann cells were firstly seeded at a density of 5 × 10^4^ cells per well in 48-well cell-culture plates. After cell attachment, CA@Exos-MNs were gently covered onto the cells and incubated for 1.5 h. Subsequently, an ES with a potential of 400 mV was applied for 30 min using a home-made high throughput device. Control groups received identical treatment without ES. After treatment, the medium was removed, and 300 μL of serum-free Dulbecco's modified Eagle's medium (DMEM) supplemented with 30 μL of CCK-8 solution was added to each well. After incubation at 37 °C for 2 h, absorbance of each well at 450 nm was measured using a microplate reader. For each group, there were 5 parallel samples.

**Cumulative release of** **CA@Exos from CA@Exos-MNs in vitro**

CA@Exos-MNs were placed in 1 mL of PBS in a shaker (HNY-200F) at 37 °C. At predetermined time intervals (24 and 48 h), 50 µL of the supernatant was withdrawn and replenished with an equal volume of fresh PBS. The content of released CA@Exos in supernatant was measured by using a BCA protein quantification kit. Three parallel samples were used in the test. The cumulative release of CA@Exos was calculated by the equation S7:

$$\begin{aligned} The cumulative release \left( \% \right)=\left( V_{e}\sum_{1}^{n-1} C_{i}+V_{o}C_{n} \right)/m\times100\#(Equation S7) \end{aligned}$$

Where V_e_ represents the volume of withdrawn solution; C_i_ represents the concentration of CA@Exos at each timepoint; V_0_ represents the volume of initial PBS; C_n_ represents the concentration of CA@Exos at the first time point (24 h); m represents the amount of CA@Exos on CA@Exos-MNs.

**Porcine skin insertion ability of** **CA@Exos- MNs**

DiO-labeled-CA@Exos-MNs were vertically pressed on porcine skin purchased from a local market in Qingdao for 20 min. The porcine skin was then visualized using CLSM (TCS SP8 STED 3X), where cross-sectional images were acquired at 10 µm intervals to evaluate the distribution of DiO-labeled-CA@Exos at various skin depths.

**Biodistribution of CA@Exos in vivo**

The CA@Exos were labeled using 1,1'-dioctadecyl-3,3,3',3'-tetramethylindodicarbocyanine,4-chlorobenzenesulfonate salt (DiD) following the manufacturer’s instructions. The DiD-labeled-CA@Exos (200 µg) was incubated with MNs (8 × 8 mm) for 12 h at 4 °C to allow loading. Balb/c mice (female, 4 weeks) were obtained from Jinan Pengyue experimental animal breeding Co., Ltd (Jinan, China). Ocean University of China Animal Laboratory Animal Ethics Committee (approval number: OUC-SMP-2025-02-12) approved all animal procedures in the experiment. Animals were housed under standard conditions (22 ± 2 °C, 55 ± 5% humidity, 12 h light/dark cycle) with ad libitum access to food and water. After the mice were anesthetized with chloral hydrate (10%, 3 mL kg^-1^), the hair on the dorsum was shaved and one full-thickness circular wound (diameter: 10 mm) was created on the dorsum of mice. Then, the DiD-labeled-CA@Exos-MNs were inserted to the wound area of the Balb/c mice, followed by monitoring using a Tanon ABL-X5 imaging system (Shanghai Tanon Life Science Co.,Ltd) at 6, 24, and 48 h post-inserting. Animals were euthanized by overdose of anesthetics followed by cervical dislocation.

**In vivo evaluation of CA@Exos-MNs combined with ES treatment for full-thickness wound healing in diabetic rats**

To evaluate the in vivo therapeutic effects of CA@Exos-MNs combined with ES, a diabetic rat model with full-thickness wounds was established, with permission from Ocean University of China Animal Laboratory Animal Ethics Committee (approval number: OUC-SMP-2024-03-08). 18 Sprague Dawley (SD) rats (male, ~200 g) were obtained from Jinan Pengyue experimental animal breeding Co., Ltd (Jinan, China). Animals were housed under standard conditions (22 ± 2 °C, 55 ± 5% humidity, 12 h light/dark cycle) with ad libitum access to food and water. In brief, streptozotocin (55 mg kg^-1^ in citrate buﬀer solution) was injected intraperitoneally into rats for 3 days and the blood glucose levels were monitored. A random concentration of blood glucose above 16.7 mmol L^-1^ were considered indicative of diabetes in rats. For comparison, SD rats in the control group received intraperitoneal injections of PBS for 3 days. Both body weight and blood glucose levels were monitored throughout the process. After the rats were anesthetized with chloral hydrate (10%, 3 mL kg^-1^), the hair on the dorsum was shaved and four full-thickness circular wounds (diameter: 10 mm) were created on the dorsum of each diabetic rat. The rats were randomly divided into 8 groups, including 1) no treatment (Blank, used as control), 2) Exos, 3) MNs, 4) CA@Exo-MNs, 5) only electrical stimulation (ES), 6) Exos + ES, 7) MNs + ES, 8) CA@Exo-MNs + ES, and 9) free CA solution (31 μL, 0.1 mg mL^-1^, same CA dosage as delivered by the CA@Exos). Wound healing progression was recorded by photographing the wounds on days 0, 2, 4, and 8 post-treatment. Before implantation, CA, Exos and CA@Exos solution were sterilized by filtration through a 0.22 μm syringe-driven filter unit. MNs were sterilized by UV irradiation under 254 nm for 12 h. For the ES groups, the electrical signal (AC, 12 V, 0.1 Hz) was applied by an electrical stimulation device (JCE-203, SOSTONE, China), and the ES treatment was 60 min per day on the first 8 days after implantation. The Exos, MNs, and CA@Exos-MNs were applied every two days for four consecutive times. All wounds were photographed on day 2, 8, 14, 21, and 28 post-wounding for wound closure evaluation. Animals were euthanized by overdose of anesthetics followed by cervical dislocation. The wound area was measured through Image J software (v1.53k) by tracing the wound boundaries of the photographs. The wound healing ratio (%) was calculated as following equation S8:

$$\begin{aligned} Wound healing ratio \left( \% \right)=\frac{A_{0}{-A}_{t}}{A_{0}}\times100\#(Equation \end{aligned}S8)$$

where A_t_ is the wound area observed at 2, 8, 14, 21, and 28 days, A_0_ is the initial wound area at 0 day.

**Histological staining and immunofluorescence staining**

On day 2, 14, and 28, wound tissues were collected and fixed in 4% paraformaldehyde. After being embedded in paraffin and sliced into sections, hematoxylin and eosin (H&E) staining was performed and the sections were imaged with a digital slide scanner (Pannoramic DESK, Hungary). Furthermore, the immunofluorescence staining of CD68 (ab303565, Abcam, USA), CD86(bs-1035R, BIOSS, China), and CD206 (24595, CST, USA) was performed on tissue sections collected on day 2. The immunofluorescence staining of AGEs (HY-P81087) was performed on tissue sections collected on day 14. The immunofluorescence staining of endothelial cell adhesion molecule-1 (CD31, GB300604, Servicebio, China), α-smooth muscle actin (αSMA, GB111364-100, Servicebio, China), neurofilament 200 (NF200, GB12143, Servicebio, China), Protein gene product 9.5 (PGP9.5, GB15159, Servicebio, China), and Cytokeratin 14 (CK14, GB15803, Servicebio, China) was performed tissue sections collected on day 28. Cell nuclei were stained with DAPI. The immunofluorescence images were observed under a fluorescent microscope (Pannoramic 250 FLASH, 3DHISTECH, Hungary). The fluorescence area was quantiﬁed by Image J software (v1.53k).

**Transcriptomic evaluation of** **CA@Exos-MNs + ES treatment for diabetic wound healing**

The wound tissue samples in Blank and CA@Exos-MNs + ES groups were collected on day 28 for RNA sequencing experiments. Briefly, total RNA was extracted from the skin tissue using Trizol reagent. The RNA purity and concentration were measured using a Qubit 4.0 fluorescence quantifier (Thermo Fisher Scientific, USA). RNA integrity was assessed using a Qsep400 Bioanalyzer (BiOptic Inc., China). Subsequently, cDNA libraries were constructed and sequenced on the Illumina platform by Metware Biotechnology Co., Ltd. (Wuhan, China). Differentially expressed genes were analyzed using DESeq2 software (version 1.38.3).

**Statistical Analysis**

Statistical analysis was conducted by using GraphPad Prism software 10.4.1. All quantitative data were presented as the mean values ± standard deviation (SD). The differences between two groups were evaluated using a two-tailed, unpaired Student’s t test. The differences among multi-groups were performed using one-way analysis of variance (ANOVA) with Tukey’s multiple comparisons. Differences were considered statistically significant at *P* < 0.05.


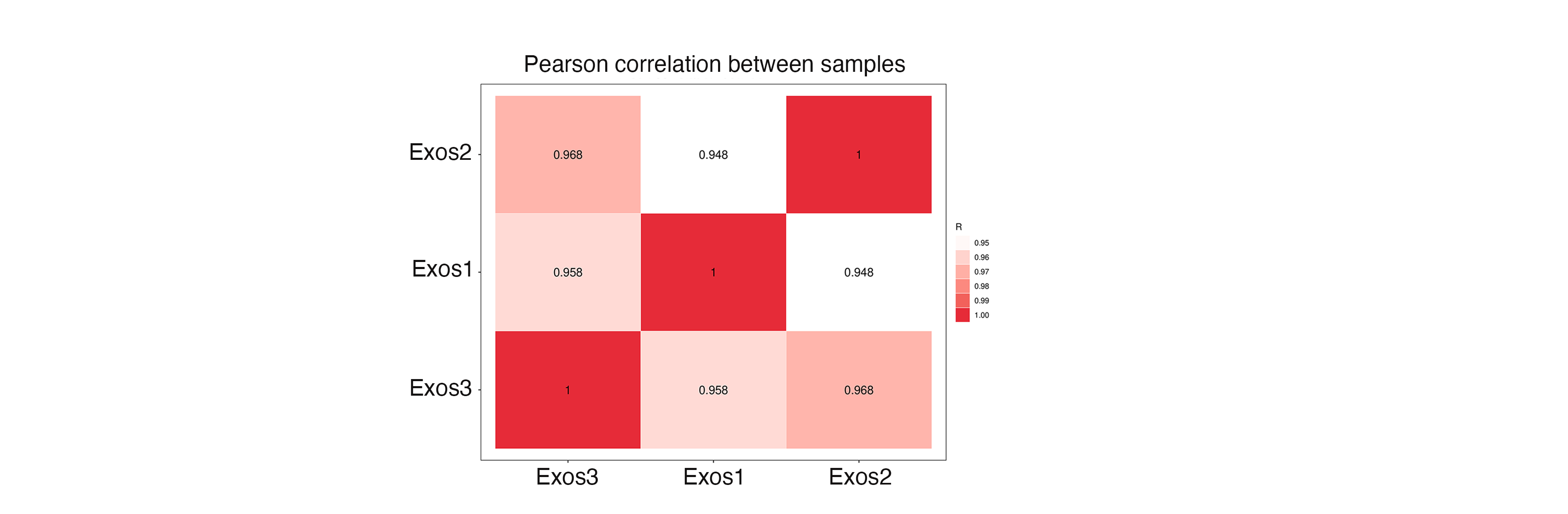


**Figure S1.** Pearson correlation between Exos derived from three distinct batches.

**
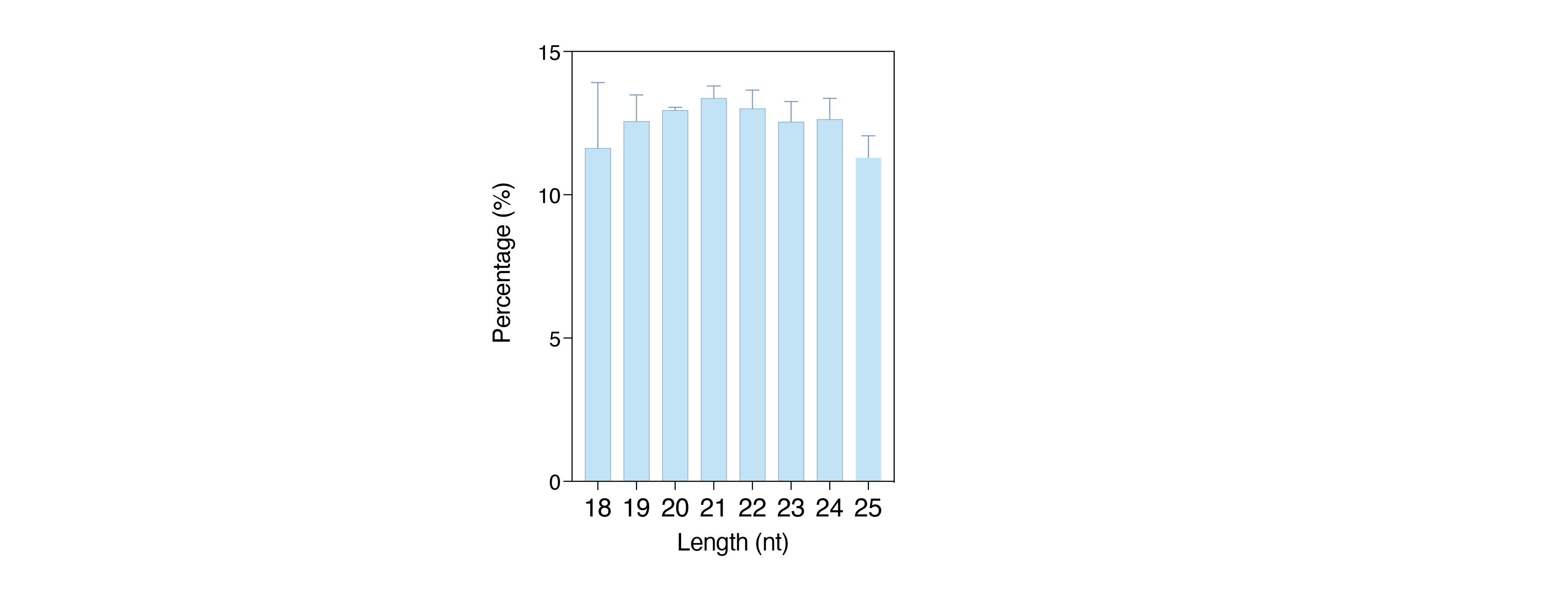
**

**Figure S2.** Length distribution of valid reads.

**
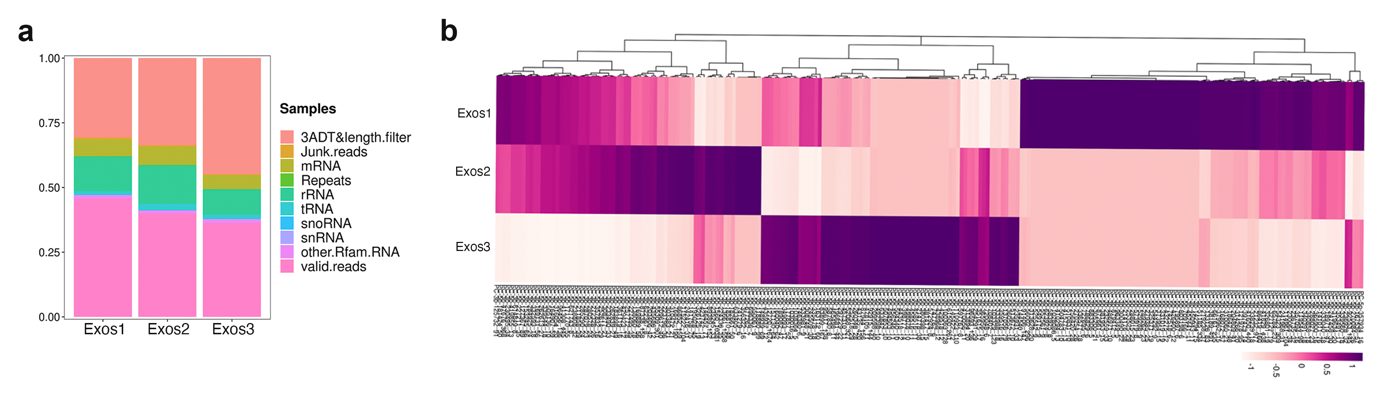
**

**Figure S3.** a) Classification of small nucleic acids contained in Exos. b) Heat map of the miRNAs in Exos derived from three distinct batches.

**
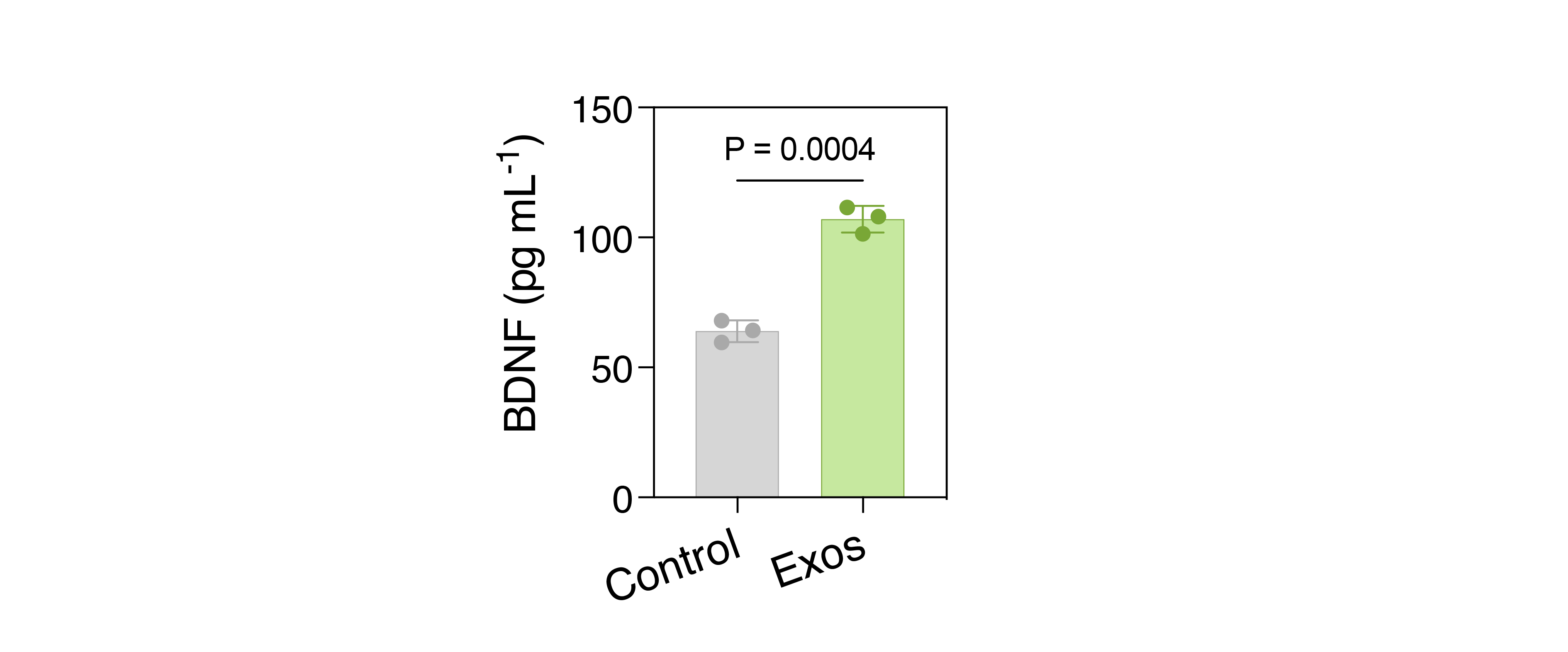
**

**Figure S4.** The expression levels of BDNF in culture medium of Schwann cells co-cultured with Exos (50 µg mL^-1^) for 6 days (*n* = 3). Data are presented as mean values ± SD. Comparisons were performed by unpaired two-tailed Student’s t test.

**
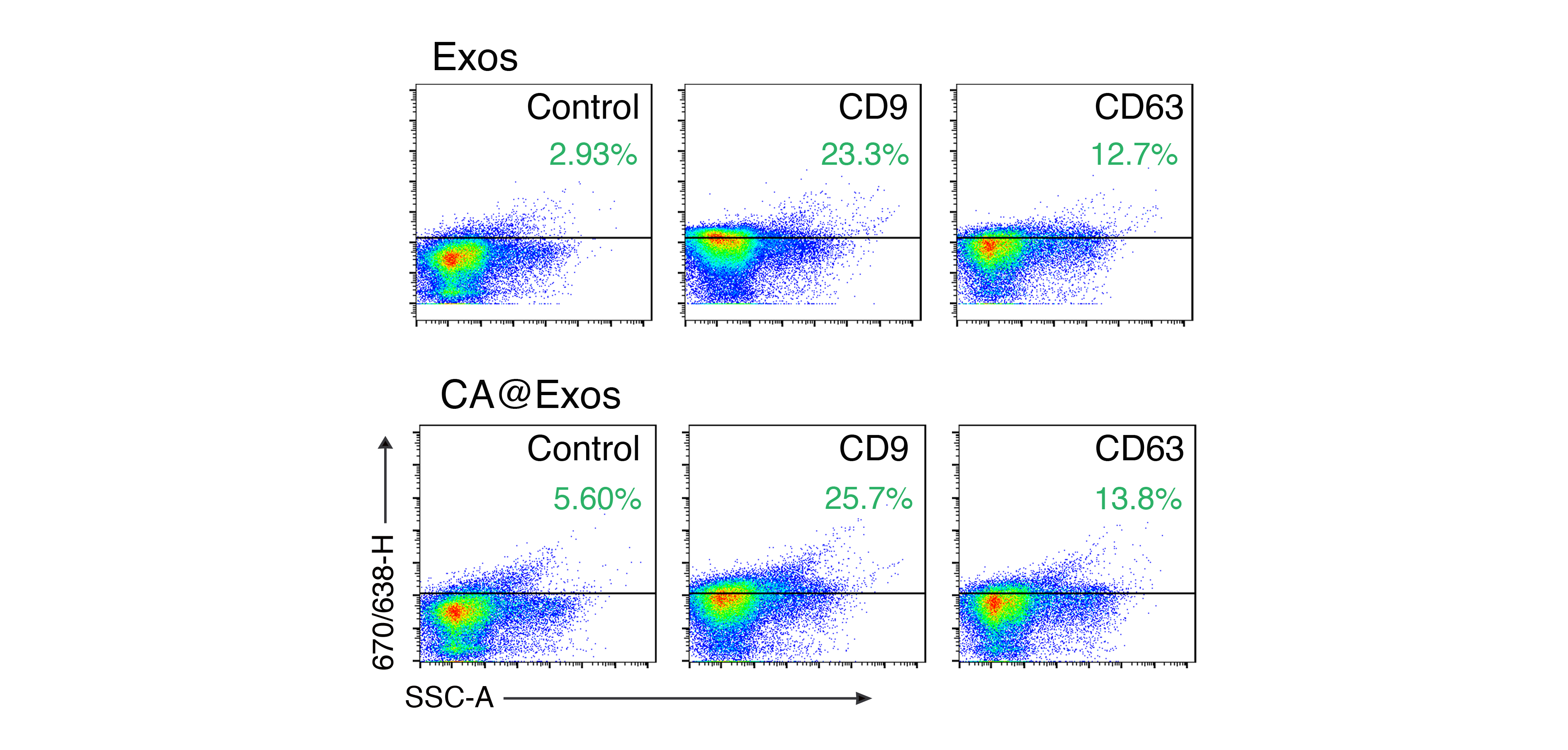
**

Figure S5. Nano-flow cytometry analysis of surface markers (CD9 and CD63) on Exos and CA@Exos.


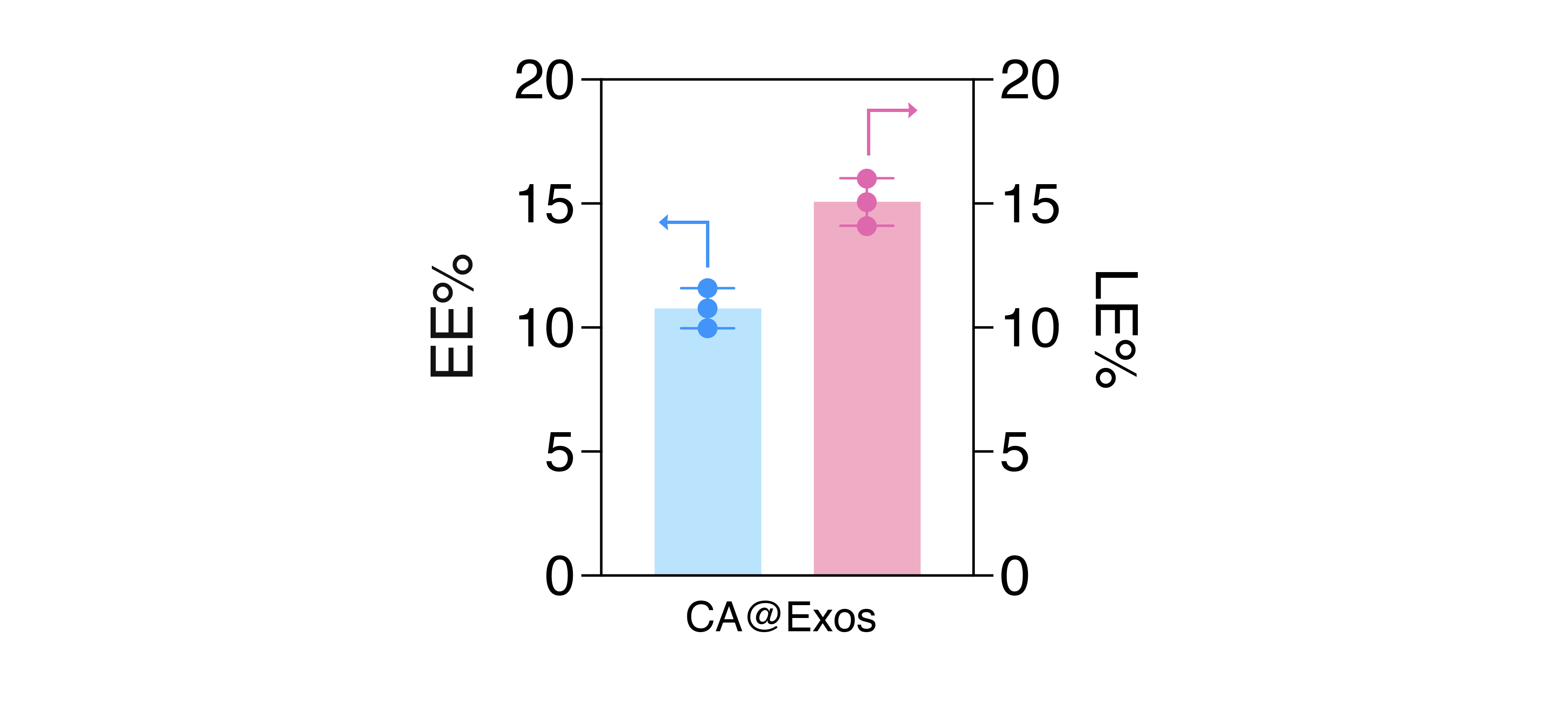


Figure S6. The encapsulating efficiency (EE) % and loading efficiency (LE) % of CA@Exos (*n* = 3). Data are presented as mean values ± SD.


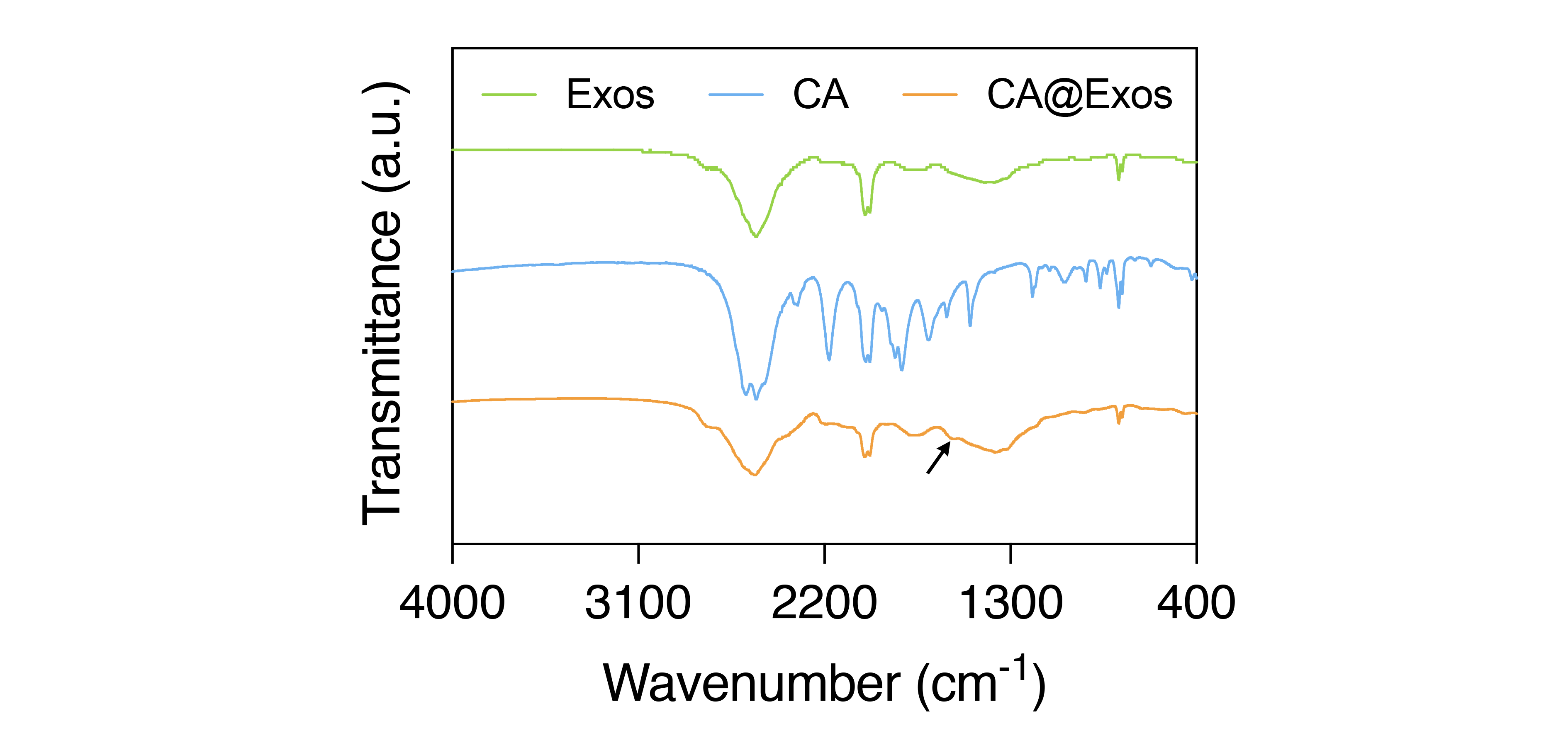


Figure S7. FTIR spectra of Exos, CA, and CA@Exos.


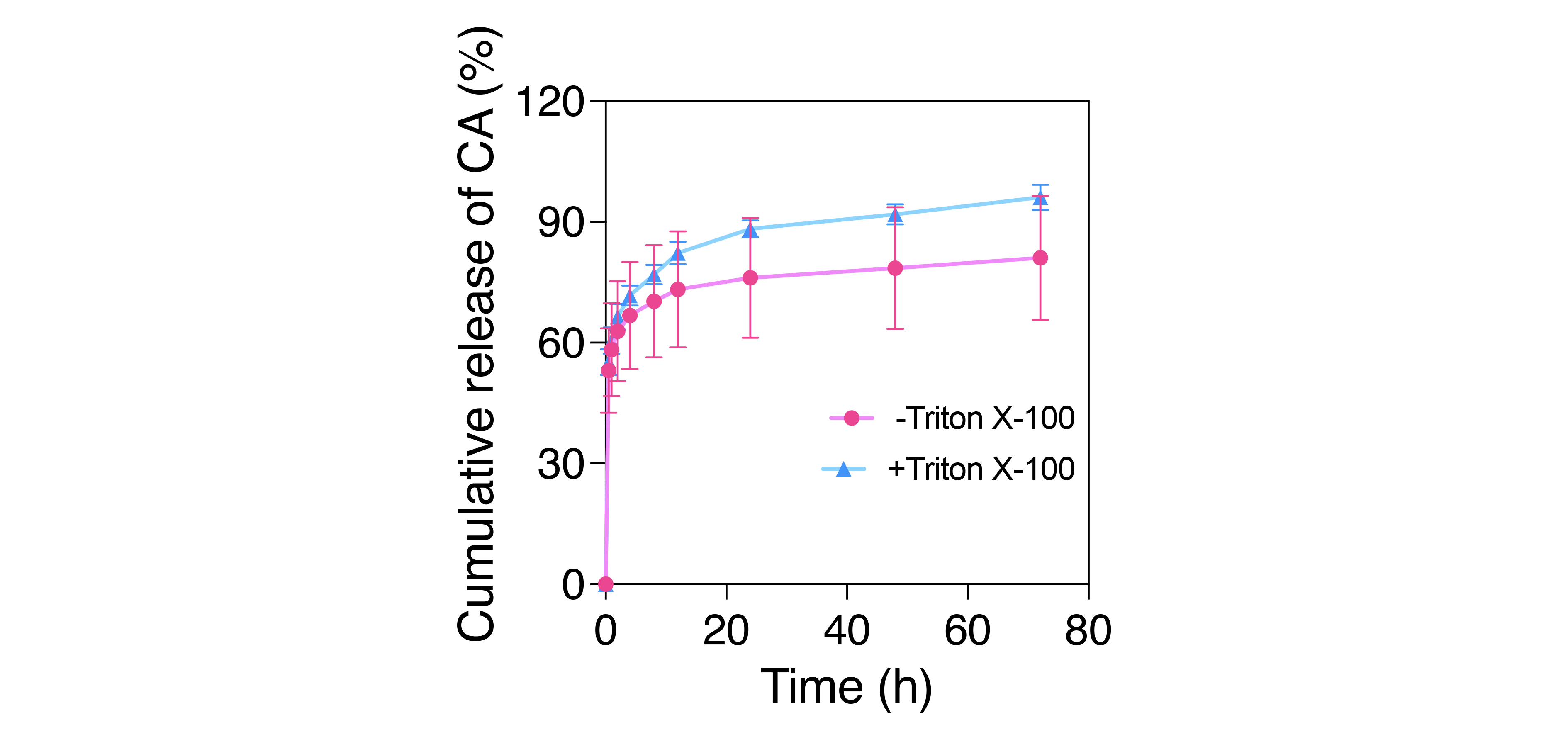


**Figure S8.** Release curve of CA from CA@Exos incubated in PBS (pH 7.4) containing 0.5% Tween 80 or PBS (pH 7.4) containing 0.5% Tween 80 and 0.1% Triton X-100 at 37 °C for 72 h (*n* = 3).


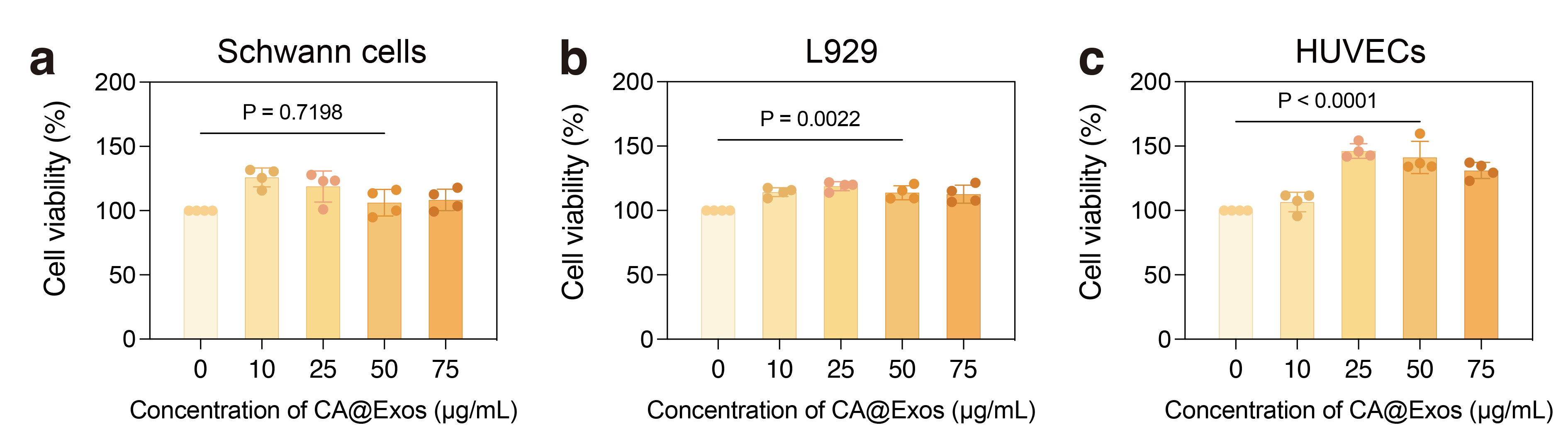


Figure S9. Cell viability assay of a) Schwann cells, b) L929 cells, and c) HUVECs co-cultured with different concentrations (0, 10, 25, 50, and 75 µg mL^-1^) of CA@Exos for 24 h (*n* = 4). Data are presented as mean values ± SD. Comparisons were performed by one-way ANOVA followed by Tukey’s multiple comparisons test.


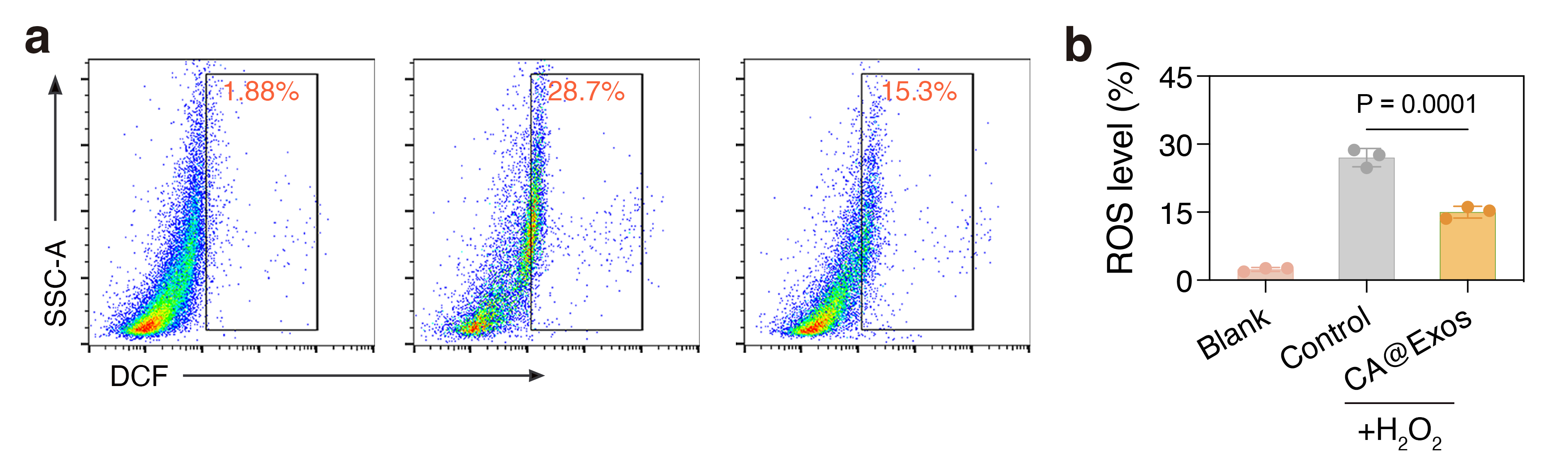


**Figure S10.** a) Flow cytometric analysis and b) corresponding quantitative analysis of ROS levels in Schwann cells with diﬀerent treatment conditions (*n* = 3). Data are presented as mean values ± SD. Comparisons were performed by one-way ANOVA followed by Tukey’s multiple comparisons test.


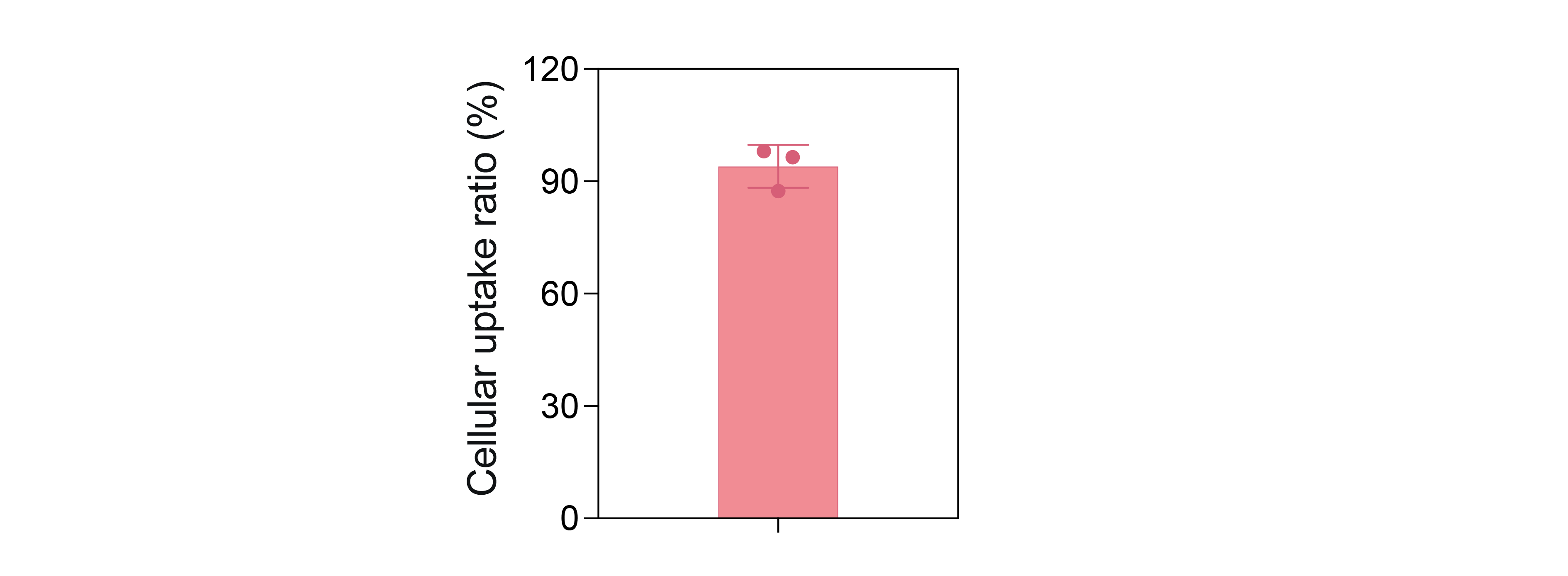


**Figure S11.** Cellular uptake ratio of DiO-labeled-CA@Exos after incubated with Schwann cells for 24 h (*n* = 3). Data are presented as mean values ± SD.


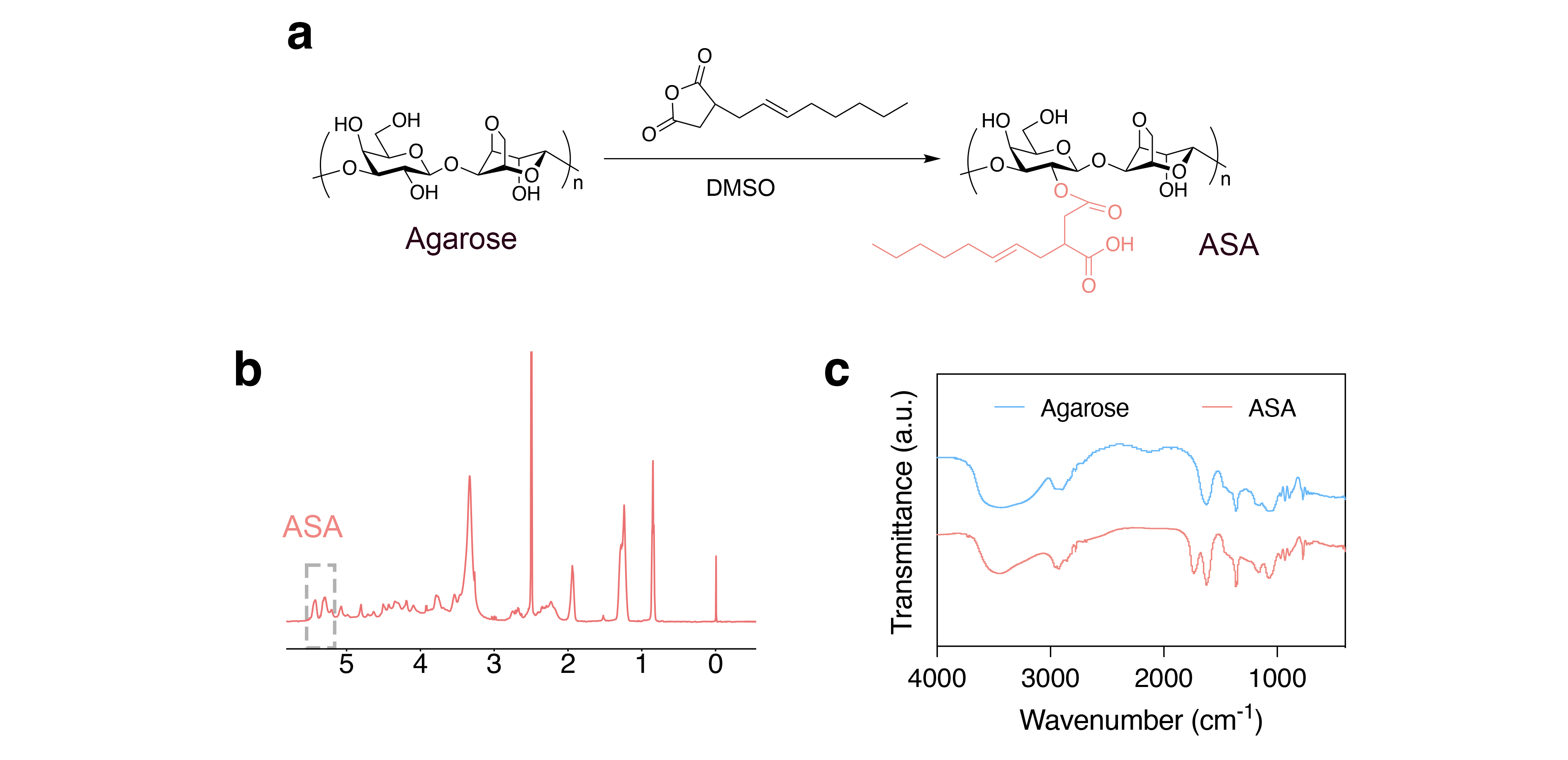


Figure S12. Characterization of ASA. a) Synthetic process of ASA. b) ^1^H NMR spectrum of ASA. c) FTIR spectra of agarose and ASA.


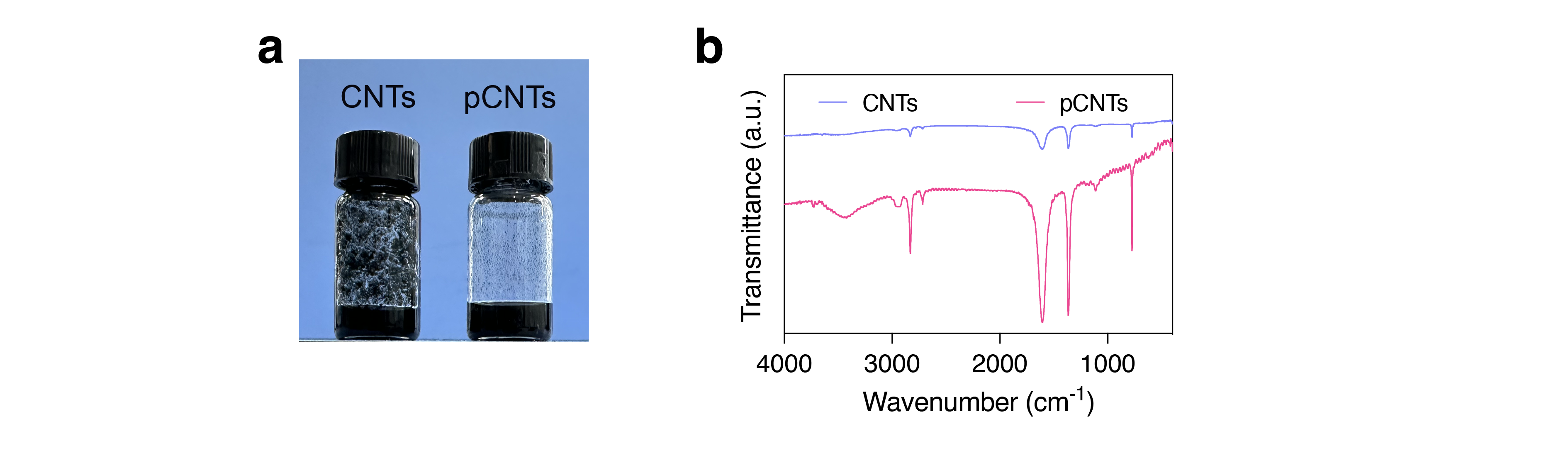


Figure S13. Characterization of pCNTs. a) Photograph showing the dispersion of CNTs and pCNTs in DMSO. b) FTIR spectra of CNTs and pCNTs.


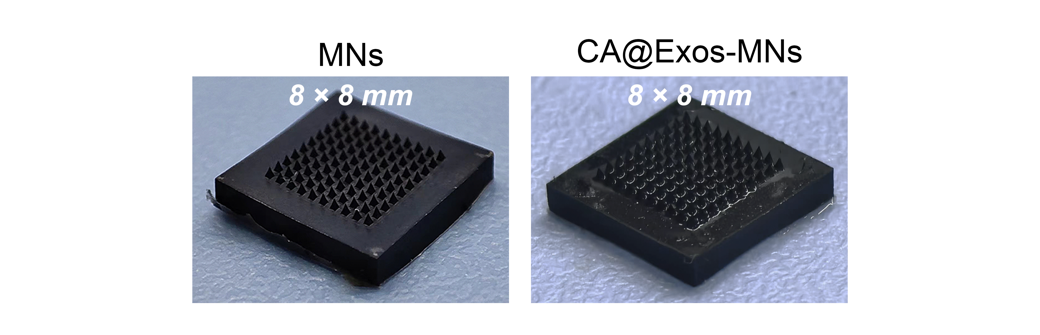


**Figure S14.** Optical photographs of the microneedle patch before and after the CA@Exos adsorption.


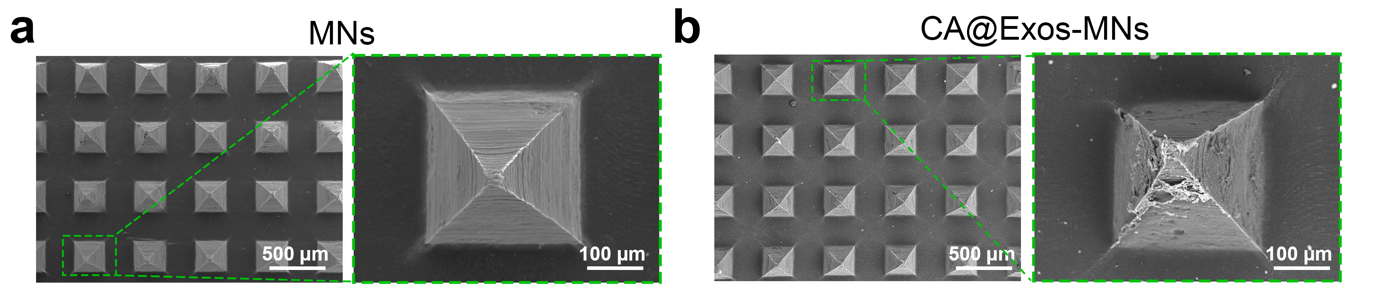
**Figure S15.** SEM images of the microneedle patch a) before and b) after the CA@Exos adsorption.


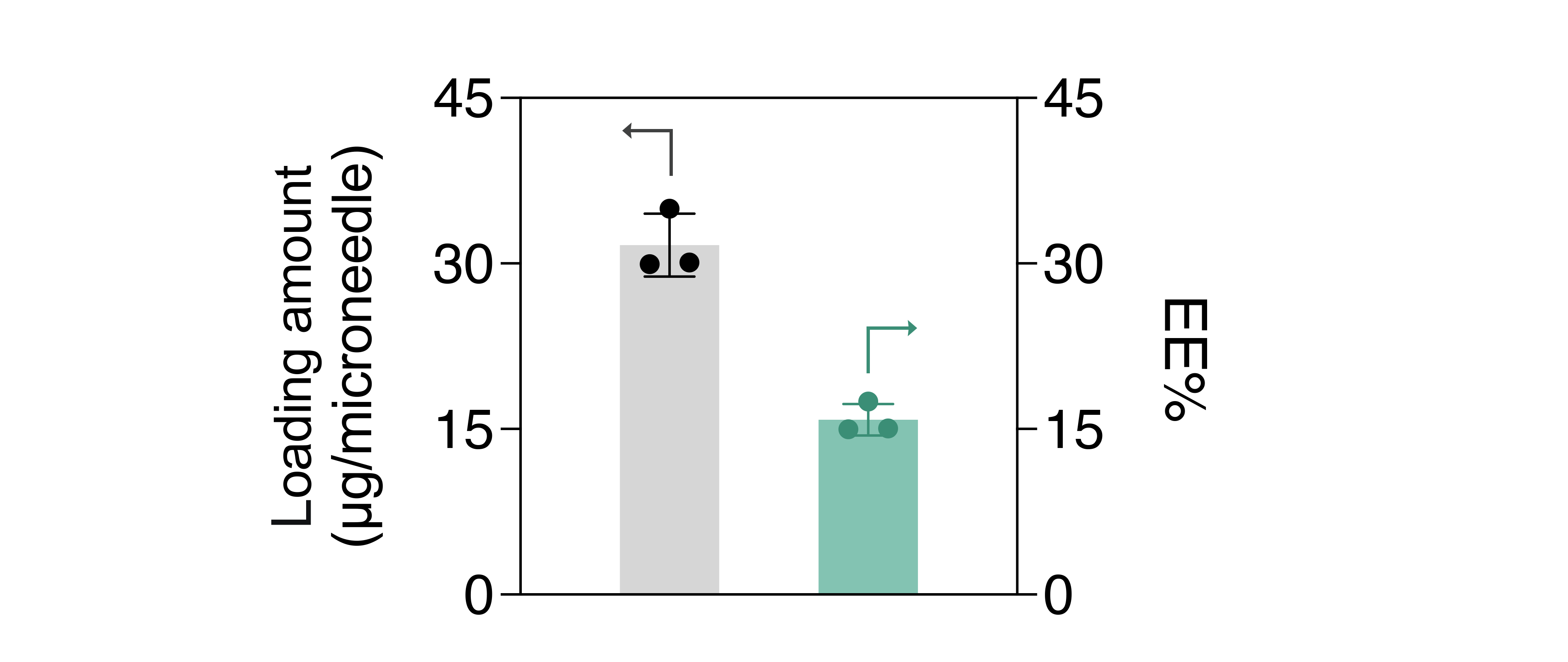


Figure S16. The loading amount of CA@Exos loaded on CA@Exos-MNs and EE% of CA@Exos-MNs (*n* = 3). Data are presented as mean values ± SD.


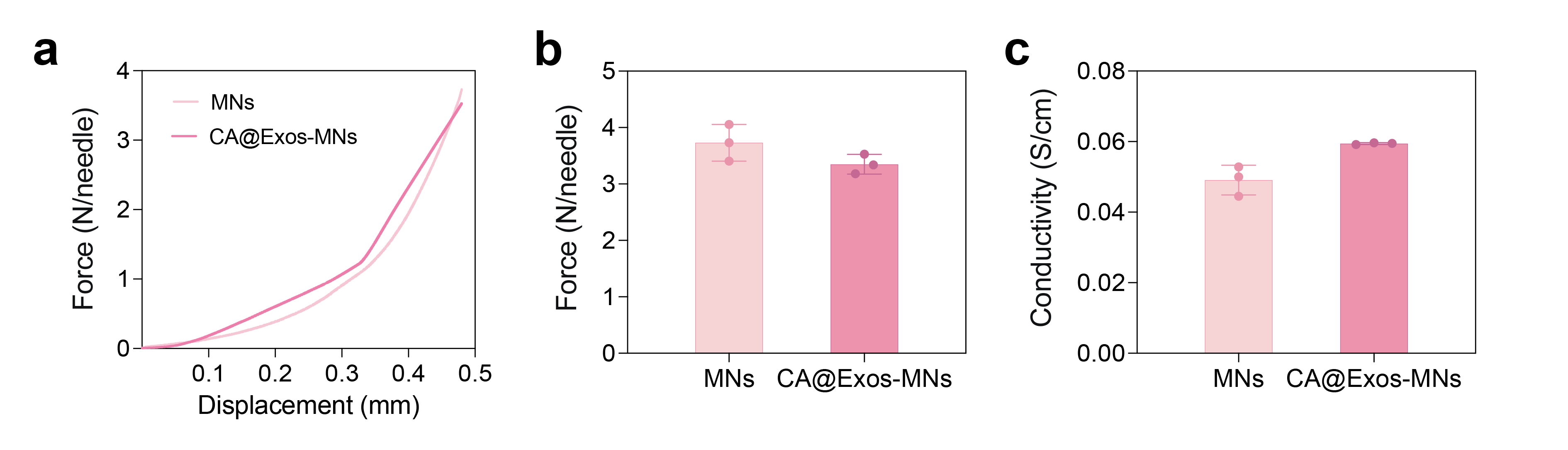
 **Figure S17.** a) The compressive force-displacement curves of MNs and CA@Exos-MNs. b) The compressive force (N per needle) of MNs and CA@Exos-MNs (*n* = 3). c) Conductivity of MNs and CA@Exos-MNs (*n* = 3). Data are presented as mean values ± SD.


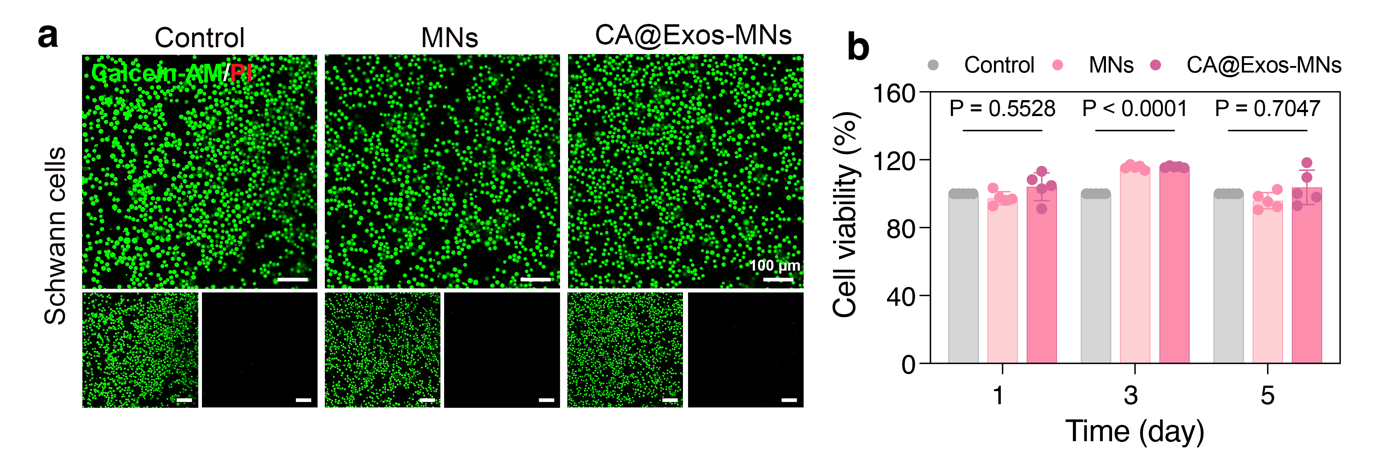


Figure S18. Cytocompatibility of MNs and CA@Exos-MNs. a) Live/dead staining of Schwann cells co-cultured with extract solution of MNs and CA@Exos-MNs (4 mg mL^-1^) for 3 days. Tissue culture plate (TCP) was used as control. b) Cell viability of Schwann cells co-cultured with extract solution of MNs and CA@Exos-MNs (4 mg mL^-1^) at 1, 3, and 5 days (*n* = 5). Data are presented as mean values ± SD. Comparisons were performed by one-way ANOVA followed by Tukey’s multiple comparisons test.

**Figure S19.** Cell viability assay of Schwann cells treated with ES (400 mV) for 30 min (*n* = 5). Data are presented as mean values ± SD. Comparisons were performed by unpaired two-tailed Student’s t test.


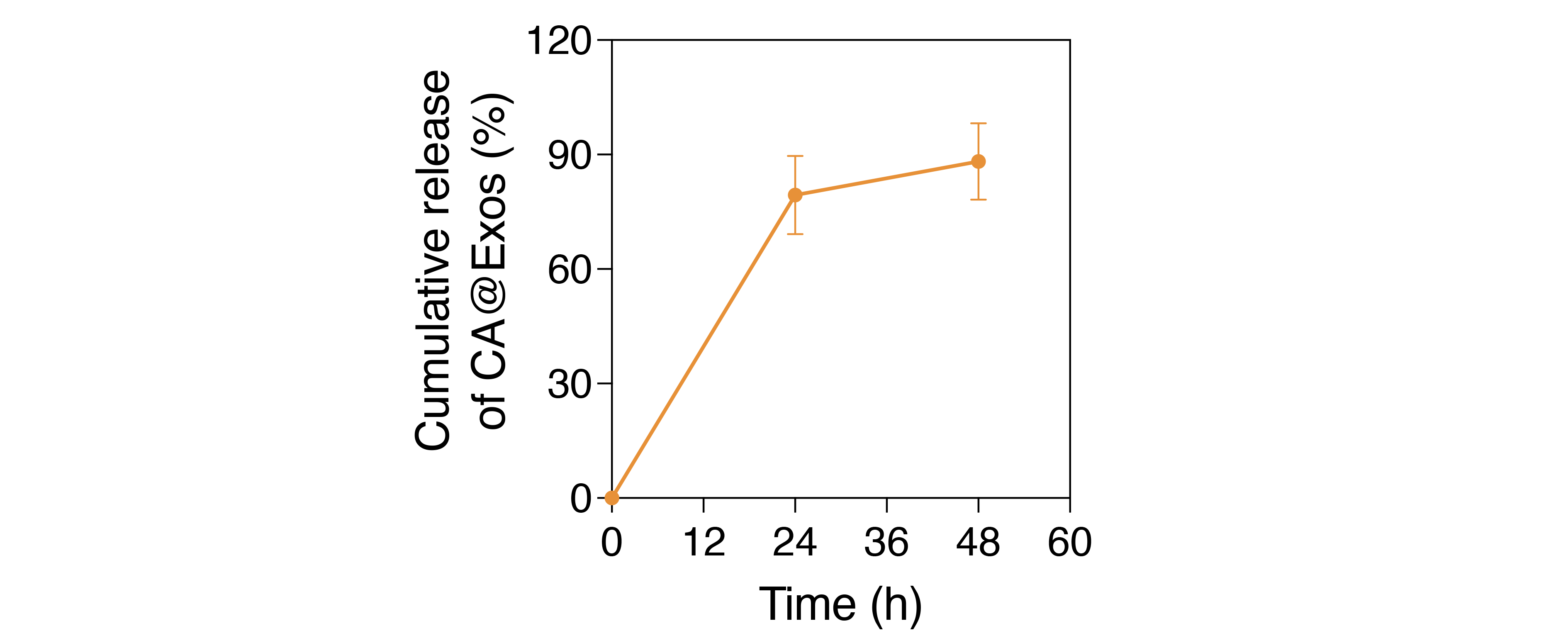


Figure S20. In vitro cumulative release curve of CA@Exos from CA@Exos-MNs within 48 h (*n* = 3). Data are presented as mean values ± SD.


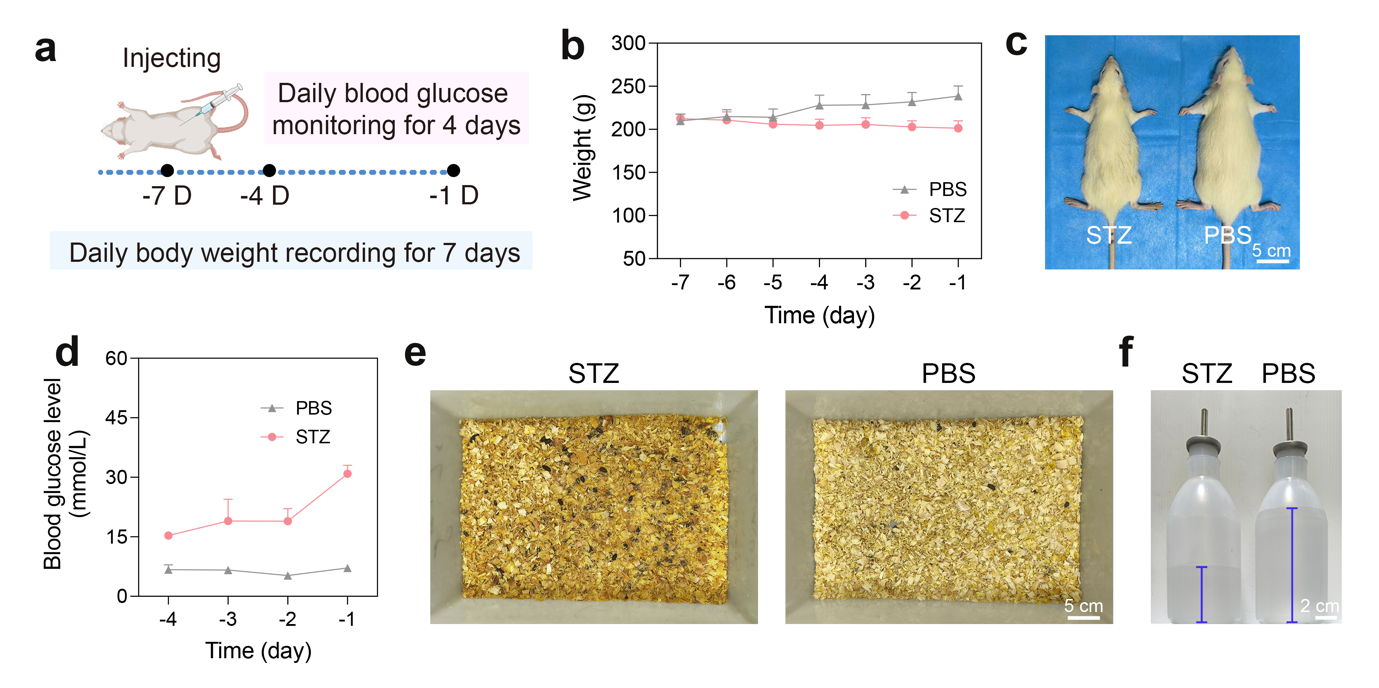


**Figure S21.** Establishment of STZ-induced diabetic SD rat model. a) Schematic schedule for the establishment of STZ-induced diabetic SD rat model. b) Body weight profiles of PBS- and STZ-injected SD rats (*n* = 3). c) Representative photograph showing the body size of PBS- and STZ-injected SD rats at -1 day. d) Blood glucose levels of PBS- and STZ-injected SD rats (*n* = 3). Photographs illustrating the e) excreta output and f) water intake in PBS- and STZ-injected SD rats at -1 day. Data are presented as mean values ± SD.

**
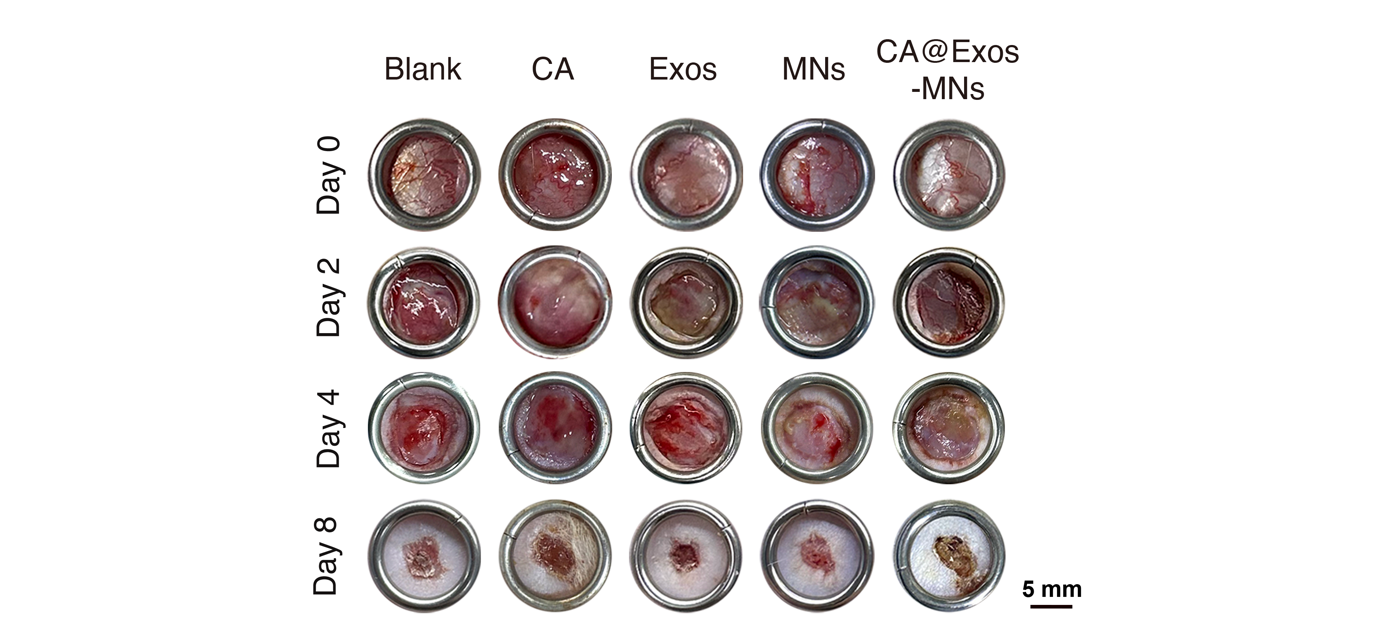
**

**Figure S22.** Representative photographs of wounds in different treatment groups on days 0, 2, 4, and 8.


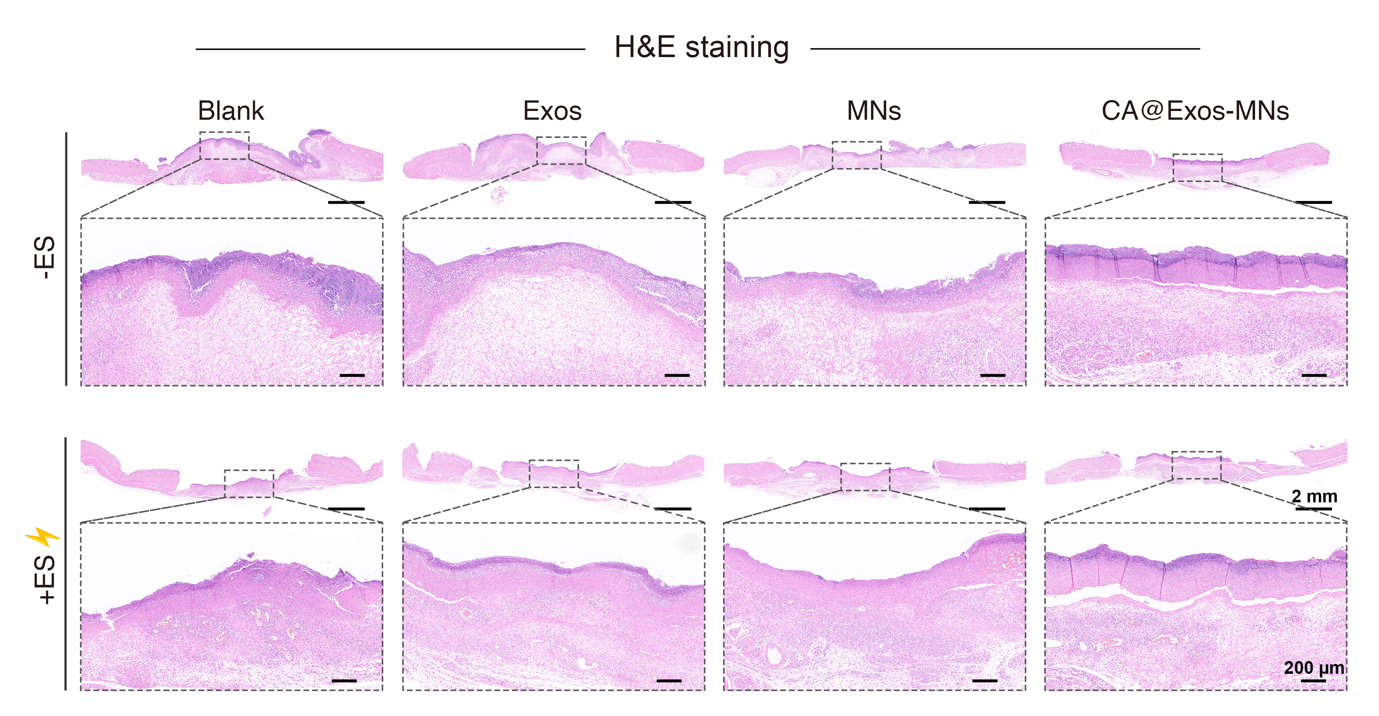


**Figure S23.** Representative images of H&E staining of tissue sections in different groups on day 2.

**Table S1.** Primers used for RT-qPCR in HUVECs.

| mRNA | Forward sequence (5’-3’) | Reverse sequence (5’-3’) |
| --- | --- | --- |
| VEGF | GGAGGGCAGAATCATCACGA | GCTCATCTCTCCTATGTGCTGG |
| GAPDH | GGAAGCTTGTCATCAATGGAAATC | TGATGACCCTTTTGGCTCCC |

**Table S2.** Primers used for RT-qPCR in Schwann cells.

| mRNA | Forward sequence (5’-3’) | Reverse sequence (5’-3’) |
| --- | --- | --- |
| BDNF | CTCTGCTCTTTCTGCTGGA | TATCTGCCGCTGTGACC |
| S100β | GGTGACAAGCACAAGCTGAA | TGGAGACGAAGGCCATAAAC |
| NGF | TGATCGGCGTACAGGCAGA | GAGGGCTGTGTCAAGGGAAT |
| GAPDH | CTGGAGAAACCTGCCAAGTATG | GGTGGAAGAATGGGAGTTGCT |

**Table S3.** Primers used for RT-qPCR in HUVECs

| mRNA | Forward sequence (5’-3’) | Reverse sequence (5’-3’) |
| --- | --- | --- |
| CD31 | ACCAAGATAGCCTCAAAGTCGG | CTGGGAGAGCATTTCACATACG |
| VEGF | GGAGGGCAGAATCATCACGA | GCTCATCTCTCCTATGTGCTGG |
| GAPDH | GGAAGCTTGTCATCAATGGAAATC | TGATGACCCTTTTGGCTCCC |

**Table S4.** Varied pCNTs/ASA ratios for preparing pCNTs-ASA MNs.

|  | pCNTs  (mg) | ASA  (mg) | pCNTs/ASA  weight ratio (wt.%) |
| --- | --- | --- | --- |
| 0 wt.% pCNTs-ASA MNs | 0 | 80 | 0 |
| 2.5 wt.% pCNTs-ASA MNs | 2 | 80 | 2.5 |
| 5 wt.% pCNTs-ASA MNs | 4 | 80 | 5 |
| 7.5 wt.% pCNTs-ASA MNs | 6 | 80 | 7.5 |
| 10 wt.% pCNTs-ASA MNs | 8 | 80 | 10 |

**Reference**

[1] S. Awasthi, N. A. Murugan, N. T. Saraswathi, Mol. Pharm. **2015**, 12, 3312.
